# PJA2 ubiquitinates the HIV-1 Tat protein with atypical chain linkages to activate viral transcription

**DOI:** 10.1038/srep45394

**Published:** 2017-03-27

**Authors:** Tyler B. Faust, Yang Li, Gwendolyn M. Jang, Jeffrey R. Johnson, Shumin Yang, Amit Weiss, Nevan J. Krogan, Alan D. Frankel

**Affiliations:** 1 Tetrad Program, Department of Biochemistry and Biophysics, University of California, San Francisco, San Francisco, California, USA; 2 Department of Biochemistry and Biophysics, University of California, San Francisco, San Francisco, California, USA; 3 Department of Cellular and Molecular Pharmacology, University of California, San Francisco, San Francisco, California, USA; 4 Department of Bioengineering and Therapeutic Science, University of California, San Francisco, San Francisco, California, USA; 5 School of Medicine, Tsinghua University, Beijing, China

## Abstract

Transcription complexes that assemble at the HIV-1 promoter efficiently initiate transcription but generate paused RNA polymerase II downstream from the start site. The virally encoded Tat protein hijacks positive transcription elongation factor b (P-TEFb) to phosphorylate and activate this paused polymerase. In addition, Tat undergoes a series of reversible post-translational modifications that regulate distinct steps of the transcription cycle. To identify additional functionally important Tat cofactors, we performed RNAi knockdowns of sixteen previously identified Tat interactors and found that a novel E3 ligase, PJA2, ubiquitinates Tat in a non-degradative manner and specifically regulates the step of HIV transcription elongation. Interestingly, several different lysine residues in Tat can function as ubiquitin acceptor sites, and variable combinations of these lysines support both full transcriptional activity and viral replication. Further, the polyubiquitin chain conjugated to Tat by PJA2 can itself be assembled through variable ubiquitin lysine linkages. Importantly, proper ubiquitin chain assembly by PJA2 requires that Tat first binds its P-TEFb cofactor. These results highlight that both the Tat substrate and ubiquitin modification have plastic site usage, and this plasticity is likely another way in which the virus exploits the host molecular machinery to expand its limited genetic repertoire.

The HIV-1 Tat protein enhances transcription elongation at the viral promoter by recruiting the cellular kinase P-TEFb to paused RNA polymerase II (RNAPII) complexes^1^,^2^. Once recruited, P-TEFb, composed of Cyclin T1 (CCNT1) and cyclin-dependent kinase 9 (CDK9) subunits, phosphorylates the C-terminal domain of RNAPII, facilitating the transition from transcription initiation to elongation^3^,^4^. Unlike the many DNA-binding transcription factors that directly associate with *cis* elements in promoters and enhancers^5^,^6^, Tat activates transcription by employing an RNA-binding domain to contact a nascent structured RNA, TAR, at the 5′ end of the HIV mRNA^7^. A considerable fraction of P-TEFb in the cell is sequestered in an inactive state by forming an RNA-scaffolded complex with the 7SK snRNP^8^,^9^,^10^. This inhibited complex and Tat are directly recruited to the HIV promoter, and once nascent viral RNA is synthesized, Tat and TAR competitively displace the snRNP to activate P-TEFb kinase activity^11^,^12^,^13^. Supporting this model of activation, recent genome-wide studies have demonstrated that P-TEFb-7SK snRNP occupies the majority of promoters containing paused RNAPII^14^,^15^. Furthermore, the splicing protein, SC35, is also enriched at cellular promoters and has been found to release the 7SK snRNP from P-TEFb in a nascent RNA-binding step^14^.

Tat is regulated by several post-translational modifications that greatly expand the activity of this small viral protein. A series of acetyl transfer reactions control the RNA-binding activity of Tat, where acetylation of Lys28 by P300/CBP-associated factor (PCAF) increases the affinity of the Tat-Cyclin T1-TAR complex^16^ and acetylation of Lys50 by p300 disassembles this ternary complex^17^. Tat is also a substrate for methylation^18^,^19^ and ubiquitination^20^.

Ubiquitination is the process whereby the 76 amino acid protein ubiquitin becomes covalently attached via its C-terminus to a primary amine on a substrate protein. This reaction is carried out by an enzyme cascade composed of an E1 ubiquitin-activating enzyme, an E2 ubiquitin-conjugating enzyme, and an E3 ubiquitin ligase, which generally provides substrate specificity^21^. With over 600 E3 ligases targeting diverse substrates^21^, ubiquitination is a major signaling pathway in the cell. In addition to substrate modification with a single ubiquitin, termed monoubiquitination, a polyubiquitin chain can be generated when ubiquitin molecules are covalently linked generally through one of the seven ubiquitin lysines. Several distinct polyubiquitin linkages are associated with unique signaling outputs, with Lys48-linked chains functioning as a general degradation signal and Lys11 linkages serving as a mitosis-specific degradation signal, while Lys63-linked chains often having positive signaling roles^22^. Lys11, Lys48, and Lys63 are the most abundant and well-studies linkages in the cell^23^, therefore the generalized function of the remaining ubiquitin linkages and the ligases that generate them is currently unclear.

Ubiquitin signaling has many regulatory functions in transcription^24^,^25^. One well-studied example is the mono-ubiquitination of histone H2B, which is associated with transcriptionally active chromatin regions and regulates subsequent histone modifications^26^. The tumor suppressor, p53, and proto-oncogene, Myc, are both tightly regulated by numerous ubiquitin ligases that modify multiple lysine residues in each protein. Ubiquitin modification of these transcription factors can lead to proteasomal degradation^27^,^28^,^29^,^30^, protein stabilization^31^,^32^, or protein re-localization^33^,^34^. This diversity of functional outputs makes ubiquitination an attractive means to expand the function of a viral protein as it hijacks the host cell machinery, and especially to enhance the role of Tat in regulating transcription.

In the current study, we describe a candidate RNAi screen of previously identified high-confidence Tat interacting proteins^35^ and report that several proteins from ubiquitin signaling pathways are essential for Tat activity. One of these, PJA2, is a RING finger E3 ligase that polyubiquitinates Tat. Interestingly, these modifications are non-degradative and rather serve to regulate the transcriptional activity of Tat. Furthermore, Tat ubiquitination by PJA2 is an incredibly diverse signal, both at the substrate level, where multiple Tat lysines can function as ubiquitin acceptor sites, and at the level of the modification itself, where polyubiquitin chains can be generated through multiple linkages. This immense signal plasticity likely allows the virus to potently activate transcription in the face of a high mutation rate.

## Results

### A targeted RNAi screen establishes a significant role of ubiquitin signaling in HIV-1 transcription

**Figure 1:**
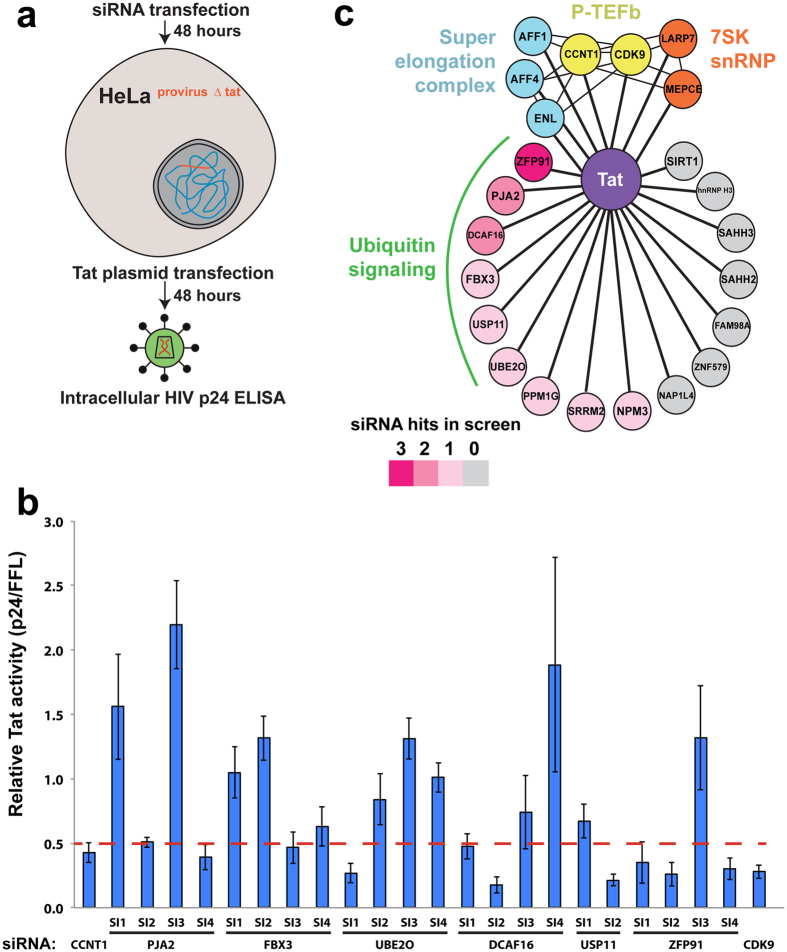
Targeted RNAi screen identifies multiple proteins involved in ubiquitin signaling as critical for Tat function. (**a**) (top) Schematic representation of the screen. A HeLa cell line containing a single, full-length integrated provirus (in red) was transfected with individual siRNAs, followed by transfection with a Tat-expressing plasmid. The readout of activity was the inhibition of Tat-dependent production of the HIV p24 protein. (**b**) Normalized, relative Tat activity ([p24^host RNAi^/FFL^host RNAi^]/[p24^N.S. RNAi^/FFL^N.S. RNAi^]) for CCNT1, CDK9, and the ubiquitin signaling proteins (PJA2, FBX3, UBE2O, DCAF16, USP11, and ZFP91). Data are represented as the mean −/+ SEM of three biological replicates for independent siRNA transfections. The red dotted line represents the activity cutoff for the screen. (**c**) Network representation of sixteen novel high confidence Tat interactors from our prior large-scale proteomics study^35^. Color-coding reflects the number of siRNAs for each factor that caused at least a 2-fold decrease in Tat activity. Also shown are known Tat interactors that are part of P-TEFb, 7SK RNP, and super elongation complexes.

Our recent proteomic interaction mapping uncovered several high-confidence host binding partners for the Tat protein^35^. However, the function of these proteins in Tat-dependent transcription remains unclear. To resolve this issue, we performed a targeted RNAi screen of sixteen high-confidence interactors in a functional assay of Tat activity. For the screen, we generated a HeLa cell line containing a single, full-length HIV provirus (Supplementary Fig. 1a) deleted for the *tat* gene (HeLa^provirusΔtat^), which allowed us precise control of transcriptional activation by transfection of a Tat-expressing plasmid (Fig. 1a). Importantly, a very small quantity of Tat plasmid that was in the linear range of activation for the HIV promoter was used in the screen, ensuring physiological Tat protein levels. The readout of the screen was the Tat-dependent production of the HIV-1 capsid (p24) protein, allowing interrogation of Tat function in a relatively natural viral context. To validate the approach and calibrate signal strength, knockdown of CCNT1 or CDK9 resulted in a greater than 2-fold decrease in p24 production compared to a non-silencing (N.S.) siRNA (Fig. 1b, Supplementary Fig. 1c for protein knockdown), effects comparable to other studies of P-TEFb knockdown on Tat activity^36^. We therefore classified siRNAs as hits if they similarly produced a 2-fold or greater decrease in p24. Of the sixteen factors tested, nine had at least one siRNA that met this threshold (Fig. 1b, Supplementary Fig. 1b). One of these, PPM1G, has recently been shown to dephosphorylate CDK9 to locally disassemble the 7SK snRNP at the HIV promoter^12^, supporting that the screen can readily identify functionally important Tat host factors. Examining the functions of these nine positives revealed an unexpected enrichment for ubiquitin signaling proteins, with three E3 ligases (ZFP91, PJA2, and UBE2O)^37^,^38^,^39^,^40^,^41^, two substrate receptors for multi-subunit E3 ligases (DCAF16, FBX3)^42^,^43^, and one de-ubiquitinating enzyme (USP11)^44^ (Fig. 1c). Thus, our functional screen of Tat interacting proteins suggested an important role for ubiquitin machinery in HIV-1 transcriptional regulation.

### The RING-H2 finger protein PJA2 ubiquitinates Tat

**Figure 2:**
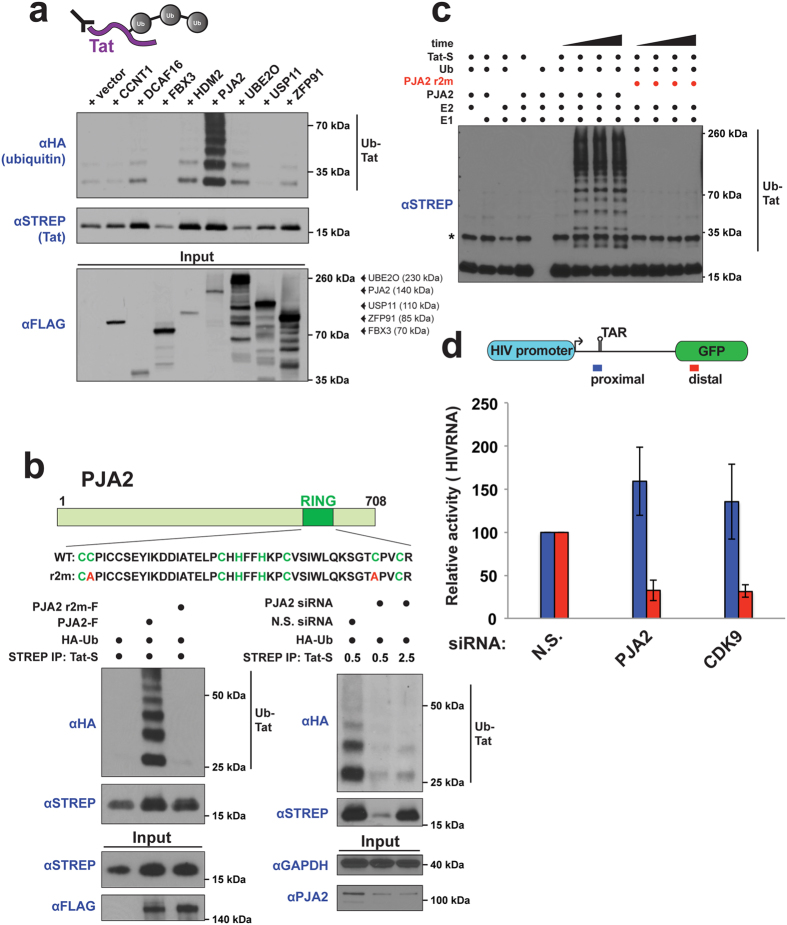
PJA2 specifically ubiquitinates Tat *in vivo* and *in vitro*. (**a**) Tat *in vivo* denaturing ubiquitination assay. HEK 293T cells were transfected with Tat-STREP, HA-Ub, and FLAG-tagged ubiquitin signaling proteins that showed activity in the RNAi screen, followed by a denaturing STREP IP of Tat (illustrated by schematic above). Tat ubiquitination was detected by anti-HA Western blotting and the ubiquitinated species are indicated. Protein sizes (in kDa) are indicated to the right of the blot. The expected size of FLAG-tagged FBX3, PJA2, UBE2O, USP11, and ZFP91 is indicated to the right of the FLAG input blot. See Sup. Fig. 2 for longer anti-HA exposures. A cropped STREP blot is shown. (**b**) (top) Domain organization of the PJA2 protein. The zinc-binding residues of the RING-H2 domain are highlighted in green and the alanine point mutations that constitute the r2m mutant are shown in red. (bottom left) *In vivo* ubiquitination assay of Tat-STREP (referred to as Tat-S) co-expressed with FLAG-tagged PJA2 wild type or RING finger mutant (r2m). A cropped STREP blot is shown for IP and input and a cropped FLAG blot for input. (bottom right) PJA2 knockdown inhibits Tat ubiquitination. The HeLa^provirusΔtat^ cell line was transfected with N.S. or PJA2 siRNA 2 (from screen), followed by transfection with HA-Ub and 0.5 ug Tat-S for N.S. RNAi cells or 0.5 ug and 2.5 ug Tat-S for PJA2 RNAi cells. *In vivo* ubiquitination of Tat is indicated by the HA blot. Western blots with anti-PJA2 show the extent of protein knockdown (~75%). A cropped STREP blot is shown for IP, while a cropped GAPDH and PJA2 blot are shown for input. For un-cropped blots, see Sup. Fig. 9. (**c**) *In vitro* ubiquitination of Tat. Tat-STREP purified from *E. coli* was incubated with recombinant E1, E2, wild type PJA2 or r2m mutant, ubiquitin, and ATP. All Tat species were detected by STREP Western blotting. A time course of ubiquitination was performed for reactions containing all components of the *in vitro* ubiquitination system (t = 0, 15, 30, 60 minutes). Reactions on the left, which were missing one component of the full *in vitro* system, were incubated for 60 minutes. The band labeled with an asterisk is not a ubiquitinated species. (**d**) HIV RNA elongation assay with PJA2 RNAi. Purified RNA was amplified with proximal and distal primer pairs as depicted in the schematic above from HeLa^provirusΔtat^ cells transfected with N.S. siRNA, PJA2 siRNA 2, or CDK9 siRNA. Data are presented as the mean −/+ SEM of relative Tat activity (indicated siRNA/N.S. siRNA) of at least 3 biological replicates. All qPCR values were normalized to RPLP0 mRNA.

Tat is known to be ubiquitinated^20^ so we surmised that one or more of these interacting, functionally important factors might be involved directly in Tat ubiquitination. To test this, we measured the ubiquitination of Tat *in vivo* upon co-expression of each of the six ubiquitin pathway proteins. We first performed these experiments in the absence of proteasome inhibitors, anticipating that non-degradative sites of ubiquitination in Tat might be used to regulate its transcriptional activity. Indeed, PJA2 co-expression dramatically increased Tat ubiquitination compared to the other candidate proteins (Fig. 2a, Supplementary Fig. 2). PJA2 is a RING-H2 E3 ligase and is a critical regulator in many kinase signaling pathways^38^,^39^,^45^,^46^. The RING domain is a commonly used E2 interaction module in E3 ligases^21^. Consistent with an important role in Tat ubiquitination, expression of a PJA2 r2m RING domain mutant (C634A, C671A) completely failed to stimulate Tat ubiquitination compared to wild type PJA2 (Fig. 2b, compare lanes 2 and 3 in left panel). Importantly, RNAi knockdown of endogenous PJA2 strongly reduced Tat ubiquitination (Fig. 2b, compare lanes 1 and 3, right panel), which, together with the co-expression experiments, demonstrate that PJA2 is a novel ubiquitin ligase for Tat. Interestingly, PJA2 can stabilize Tat protein levels, as fivefold excess of Tat plasmid was required in cells knocked down for PJA2 to yield comparable protein to non-silencing cells (Fig. 2b), consistent with Tat ubiquitination being non-degradative. Tat is a highly specific substrate, as PJA2 expression did not increase the ubiquitination of two other small HIV proteins, Rev and Nef (Supplementary Fig. 3a).

To demonstrate that PJA2 directly modifies Tat, we established an *in vitro* ubiquitination system. Using purified components, PJA2 readily ubiquitinates Tat only in the presence of ubiquitin, E1, and E2, generating high molecular weight ubiquitinated species (Fig. 2c). Importantly, the PJA2 r2m mutant did not modify Tat, supporting our *in vivo* results (Fig. 2b, left panel). To ensure the functional significance of PJA2-mediated Tat ubiquitination in regulating transcription elongation, we monitored the effect of PJA2 knockdown on the production of HIV-1 promoter-proximal and distal RNA transcripts using the HeLa^provirusΔtat^ cell line. While PJA2 knockdown did not affect promoter-proximal RNA production, it significantly decreased the level of Tat-dependent distal transcripts, an effect as potent as the knockdown of CDK9 (Fig. 2d). Thus, ubiquitination of Tat by PJA2 is a novel signal that regulates its transcriptional activity. The PJA2 protein is broadly expressed across many tissues, including the lymph nodes and tonsils of the immune system which are major sites of T cells^47^. More specifically, PJA2 is well expressed in CD4^+^ Jurkat and Sup-T1 T cells (Supplementary Fig. 3b), highlighting that this ubiquitin ligase can regulate Tat activity during natural viral infection. The additional ubiquitin pathway proteins that are critical for Tat-dependent activity (Fig. 1b) likely modulate the ubiquitination of other Tat cofactors. Supporting this idea, the UBE2O ligase ubiquitinates proteins of the 7SK snRNP to regulate the activity of this complex (accompanying manuscript). However, here we further analyzed the direct PJA2-dependent modification of this essential viral protein.

### Tat ubiquitination by PJA2 is non-degradative

All known substrates of PJA2 are targeted to the proteasome by ubiquitination^38^,^39^,^45^,^46^, however our experiments suggested that PJA2 ubiquitination of Tat is not degradative. For example, PJA2-dependent ubiquitinated Tat species are readily detectable in the absence of proteasome inhibitors (Fig. 2a), and PJA2 co-expression does not decrease steady-state Tat protein levels (Fig. 2a, STREP blot), which is characteristic of degradative ubiquitination, including previously defined PJA2 substrates. Indeed, PJA2 actually stabilizes Tat, as RNAi-mediated depletion of the ligase resulted in lower Tat protein accumulation (Fig. 2b). However, when we treated cells with the proteasome inhibitor MG132, both unmodified and ubiquitinated Tat levels increased, suggesting that Tat can be degraded by the proteasome (Supplementary Fig. 4).

**Figure 3:**
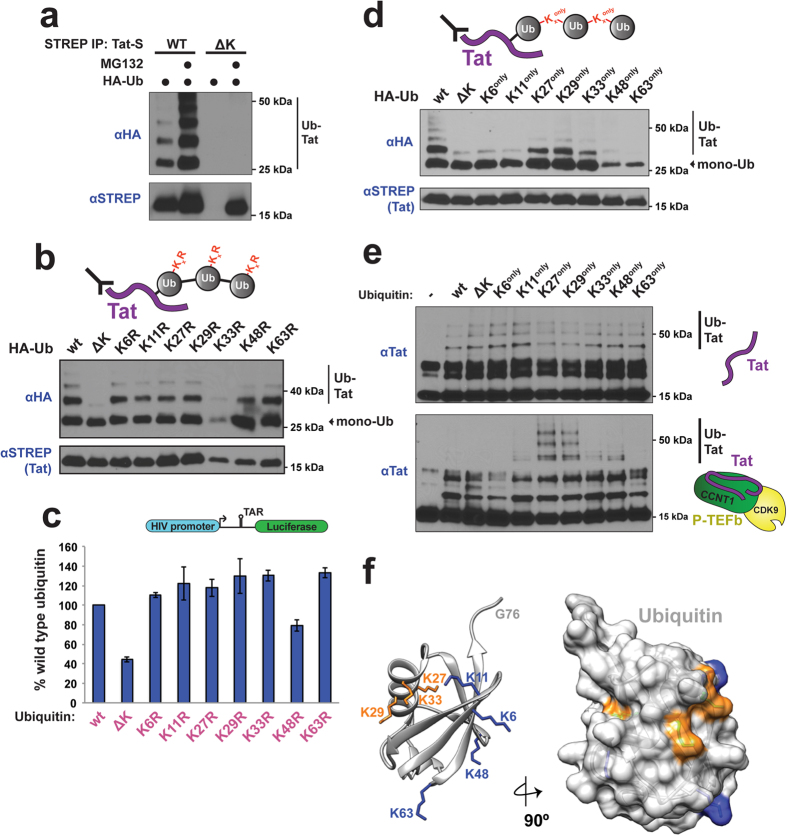
Tat is modified by PJA2 with non-degradative, atypical polyubiquitin chains. (**a**) *In vivo* ubiquitination assay of STREP-tagged (Tat-S) wild type Tat or Tat ΔK −/+ MG132 in HEK 293T cells. The anti-HA Western detects ubiquitinated Tat species. A cropped HA and STREP blot are shown. (**b**) *In vivo* ubiquitination of Tat co-transfected with wild type ubiquitin or with ΔK or individual Lys-to-Arg mutants. A schematic representation of the assay and ubiquitin mutants is shown above. Monoubiquitinated and multi-ubiquitinated species are indicated. A cropped STREP blot is shown. (**c**) (top) Schematic of the integrated luciferase reporter in the HeLa^LTR:FFL^ cell line. (bottom) Transcriptional activity of Tat after co-transfection with the ubiquitin variants used in panel (**b**). Data are represented as mean −/+ SEM of three biological replicates and are normalized to co-transfected CMV-Renilla luciferase. (**d**) *In vivo* ubiquitination of Tat co-transfected with wild type ubiquitin or the ΔK or individual Lys^only^ ubiquitin mutants. A schematic representation of the assay and ubiquitin mutants is shown above. A cropped STREP blot is shown. For un-cropped blots, see Sup. Fig. 9. (**e**) *In vitro* ubiquitination of Tat alone (top) or Tat in complex with P-TEFb (bottom) by PJA2. Wild type ubiquitin or the ΔK or individual Lys^only^ ubiquitin mutants were used and reactions were incubated for 2 hours. (**f**) (left) Ribbon representation of ubiquitin (PDB 1ubq). The lysine residues used to generate polyubiquitin chains on Tat are highlighted in orange (Lys27, Lys29, and Lys33) and the remaining ubiquitin lysines are shown in blue. (right) Surface representation of the ubiquitin structure rotated by 90° with identical coloring.

It is worth noting that MG132 is an inhibitor of the 20S core particle, which can exist free from the 26S proteasome and degrades intrinsically unstructured or oxidized proteins independently of ubiquitin conjugation^48^,^49^. Interestingly, Tat protein levels have been shown to be potently controlled by the 20S core, as small molecules that activate the 20S lead to substantial Tat degradation, an effect that is completely blocked by MG132 treatment^50^,^51^. To further support ubiquitin-independent Tat degradation, we generated a Tat mutant in which all lysine side chains were mutated to arginine (Tat ΔK) to prevent lysine ubiquitination. If Tat ubiquitination was a degradative mark, the lysine-free mutant would be expected to stabilized, as has been observed for a lysine-free Myc protein^30^. Instead, Tat ΔK protein levels were undetectable (Fig. 3a, lane 3 STREP blot), likely because mutating Lys41, which is critical for Tat folding^52^, completely destabilizes Tat and provides an unfolded substrate for ubiquitin-independent 20S core proteasomal degradation. Furthermore, Tat ΔK protein levels were fully restored upon MG132 treatment (Fig. 3a, lane 4 STREP blot). These results conclusively demonstrate that Tat protein levels are controlled by the 20S proteasome, independently of lysine ubiquitination (Fig. 3a, lanes 3–4 HA blot).

### PJA2 polyubiquitinates Tat with diverse, atypical linkages

It is generally believed that the Lys63 polyubiquitin linkage is the major signaling modality among ubiquitin systems, whereas all other linkages generally result in proteasomal degradation^53^. However some recent examples point to positive signaling functions for non-Lys63 linkages^34^,^54^. Because Tat ubiquitination is non-degradative, we wished to determine the nature of its linkage. We generated all single lysine-to-arginine mutants of ubiquitin as well as a lysine-free mutant (Ub ΔK), and co-transfected each with Tat, expecting that a point mutation would prevent addition of polyubiquitin chains through that specific residue while Ub ΔK would prevent all chain addition. As expected, Ub ΔK significantly inhibited Tat polyubiquitination (Fig. 3b, Supplemental Fig. 5a for ubiquitin mutant expression), indicating that the modified species of Tat observed are indeed polyubiquitinated and not the result of multiple monoubiquitin additions. Interestingly, no single lysine point mutant diminished Tat polyubiquitination, suggesting that diverse chain linkages can be used to modify Tat in the cell. To test if this apparent plasticity of ubiquitination linkages reflects an underlying functional plasticity, we expressed each ubiquitin mutant and measured their effects in a Tat reporter assay^11^. Correlating with the ubiquitination data, expression of Ub ΔK, and not the point mutants, strongly inhibited Tat activity (Fig. 3c), supporting a functional role for diverse polyubiquitination in Tat-mediated transcription.

This diversity of chain linkages is rather surprising for ubiquitin signaling and prompted us to test if ubiquitins engineered with single lysines (Lys^only^) could restore full Tat polyubiquitination *in vivo*. Remarkably, Lys27^only^ and Lys29^only^ variants allowed full ubiquitination of Tat and Lys33^only^ showed partial recovery (Fig. 3d, Supplemental Fig. 5b for ubiquitin mutant expression). These three lysines are contained on a single alpha helix in ubiquitin (Fig. 3f), suggesting that any one of these residues can serve as an acceptor site during chain formation on Tat. To further corroborate that this linkage diversity observed *in vivo* was being generated by PJA2, we used purified PJA2 to ubiquitinate free Tat *in vitro* and unexpectedly found no linkage preference with the single lysine ubiquitin variants, each being similar to wild type ubiquitin (Fig. 3e, top). Remarkably, however, when Tat in the context of the P-TEFb complex was used as the substrate, where Tat folds using the host proteins as a template^52^, we observed a striking preference for Lys27 and Lys29 linkages (Fig. 3e, bottom) that mirrored the linkage preference *in vivo* (Fig. 3d). These results not only highlight that the Tat-P-TEFb complex is the preferred ubiquitination substrate in the cell, but also support that PJA2 is the functional E3 ligase. Finally, using a panel of linkage-specific de-ubiquitinating enzymes (DUBs), we confirmed that Tat is modified with Lys27, Lys29, and Lys33 polyubiquitin linkages *in vivo* (Supplementary Fig. 6, YOD1 lane). Tat appears to also contain some Lys48 linkages, which might first require priming by Lys27/29/33 modification to be assembled, explaining why Lys48^only^ did not recover ubiquitination. Overall, the experiments demonstrate that Tat can be modified with diverse polyubiquitin chains containing rare linkage types by PJA2, highlighting an unanticipated activity of this host ligase, and also reveal how the substrate can exert such a large effect on the specificity of polyubiquitin acceptor site usage.

### Multiple lysine residues in Tat function as ubiquitin acceptor sites

**Figure 4:**
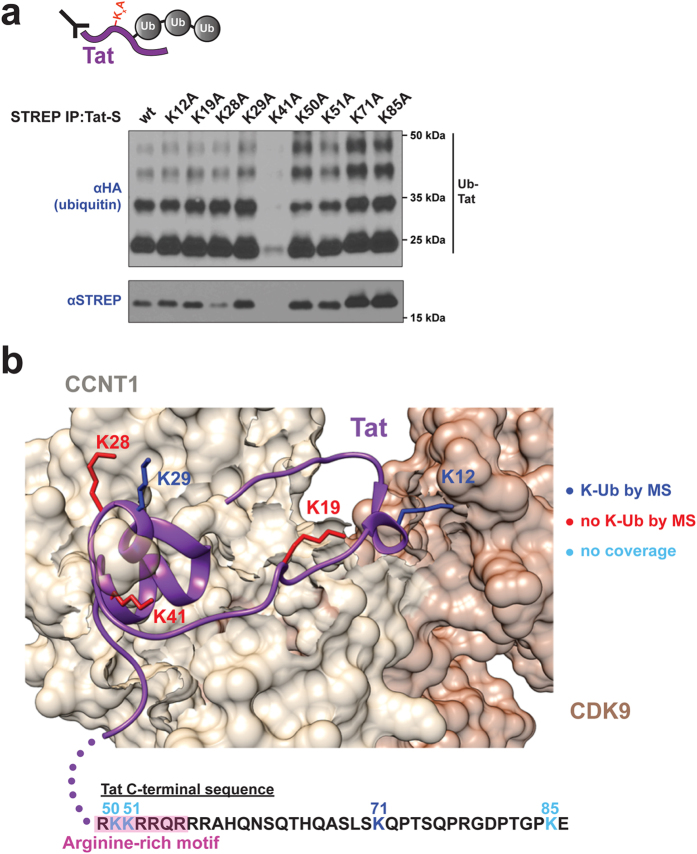
Multiple lysines in Tat can function as acceptor sites for ubiquitination. (**a**) *In vivo* ubiquitination of STREP-tagged (Tat-S) wild type Tat or individual Lys-to-Ala mutants in HEK 293 T cells. A schematic representation of the mutants is shown above. The anti-HA Western detects ubiquitinated Tat species. A cropped STREP blot is shown. For un-cropped blots, see Sup. Fig. 9. (**b**) Ubiquitinated lysines identified by MS are highlighted in blue in the Tat-P-TEFb crystal structure (PDB 3mi9) (top) or in the C-terminal Tat sequence (bottom). The color coding for modified, unmodified, or undetected lysine peptides is shown on the right.

Having shown that Tat ubiquitination is lysine-dependent (Fig. 3a), we wished to determine its ubiquitin acceptor sites. Degradative ubiquitination often displays low lysine specificity on the substrate, as the major signal is the ubiquitin chain itself, whereas non-degradative, signaling ubiquitination generally requires an additional layer of specificity in acceptor site position^55^. Given the non-degradative nature of Tat ubiquitination, we expected to find a specific site of modification. Unexpectedly, no Tat lysine-to-alanine point mutant inhibited ubiquitination (Fig. 4a), as all mutants were ubiquitinated comparably to wild type. The apparent lack of modification of K41A can be explained by low overall protein accumulation (Fig. 4a, STREP), as this mutation is known to destabilize Tat structure. This expression defect also explains why Tat ΔK was undetectable in the absence of MG132 (Fig. 3a, STREP blot).

One explanation for these results is that Tat can be ubiquitinated on multiple, redundant acceptor lysines, such that a single mutation does not inhibit ubiquitin conjugation. To explore this possibility, we used mass spectrometry (MS) to identify *in vivo* ubiquitinated side chains and detected di-glycine ubiquitin remnants on three of the nine Tat lysine residues (Lys12, Lys29, and Lys71) (Supplementary Fig. 7a,b). This result is consistent with Lys71 being previously identified as an acceptor residue^20^. It should be noted that we did not obtain full coverage of the Tat sequence by MS (Supplemental Fig. 7a), including Lys50, Lys51, and Lys85. Therefore, while we could not identify all sites of ubiquitination by MS, the results do support our model that multiple lysines in Tat can function as ubiquitin acceptor positions.

The Tat-P-TEFb crystal structure, which contains five lysines (Lys12 to Lys41) within the structured fragment of Tat, permits us to further interpret the results. Lys12 faces CDK9 in the complex but is surface-exposed and, therefore, a suitable acceptor site (Fig. 4b). Lys19 is at the interface with CycT1, which likely hinders its modification. Interestingly, of the next two adjacent, surface-exposed lysines (Lys28 and Lys29), only Lys29 is detectably ubiquitinated (Supplementary Fig. 7b). Lys28 is a known site of acetylation^16^, which may compete with its ubiquitination. Finally, Lys41 is a buried residue and is not detectably ubiquitinated (Fig. 4b). These data support a model in which multiple Tat lysines can function as acceptor residues. Together with the observed plasticity of lysine linkages in the polyubiquitin chain described above, the results suggest that an extraordinary degree of ubiquitin signaling diversity is possible on this one viral transcription factor.

### A Tat protein with only three lysines is fully functional and ubiquitinated

**Figure 5:**
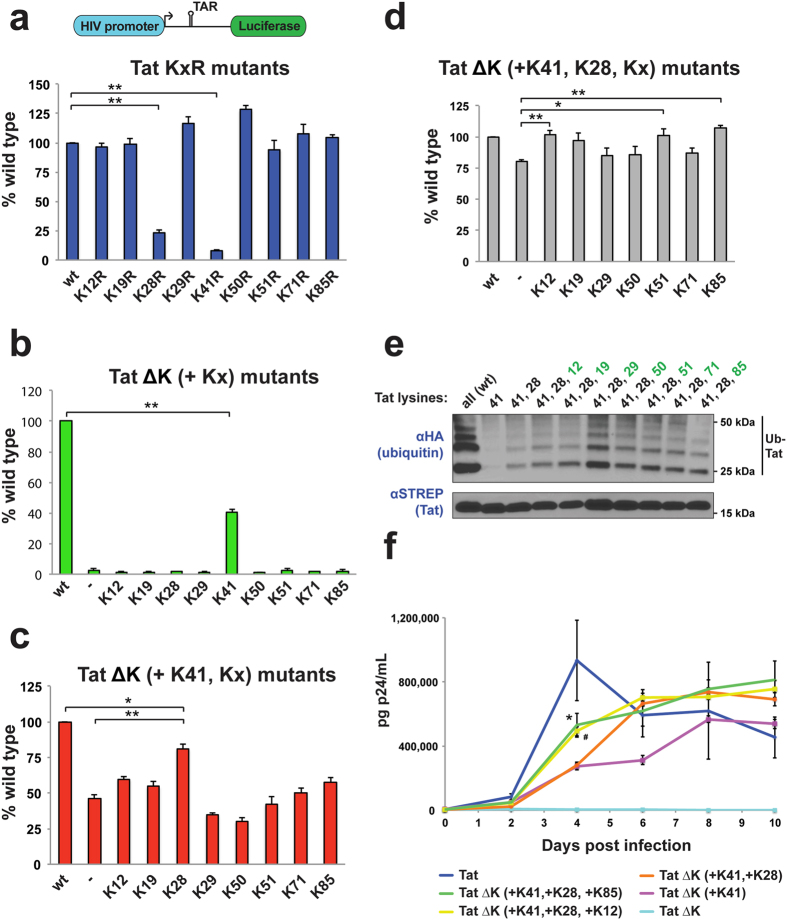
Activity and ubiquitination of minimal lysine Tat mutants. (**a–d**) Transcriptional reporter activities in the HeLa^LTR:FFL^ reporter cell line of Tat KxR mutants where individual lysines were mutated to arginine (**a**), Tat ΔK (+Kx) where individual lysines were added back to a lysine-free background (**b**), Tat ΔK (+K41, +Kx) where individual lysines were added back to a K41-only background (**c**), and Tat ΔK (+K41, +K28, +Kx) where individual lysines were added back to a K41, K28 background (**d**). Data are represented as mean −/+ SEM of at least three biological replicates, *P < 0.05, **P < 0.01. All values were normalized to co-transfected CMV-Renilla luciferase. The schematic of the integrated LTR-FFL reporter is also represented in (**a**). (**e**) *In vivo* ubiquitination assay of STREP-tagged wild type Tat or various minimal lysine mutants in HeLa cells, as indicated. A cropped HA and STREP blot are shown. For un-cropped images, see Sup. Fig. 9. (**f**) HIV spreading replication assay in Jurkat cells with either wild type Tat or minimal lysine mutants cloned into the Nef locus of pNL4-3. Data are plotted as the mean at each time point −/+ SEM (n = 4). *P < 0.05 (comparing Tat ΔK (+K41, +K28, +K85) with Tat ΔK (+K41) or Tat ΔK (+K41, +K28) at Day 4). ^#^P < 0.01 (comparing Tat ΔK (+K41, +K28, +K12) with Tat ΔK (+K41) or Tat ΔK (+K41, +K28) at Day 4). Statistical significance was determined by unpaired, two-tailed Student’s t-test.

While Tat can be ubiquitinated on multiple lysines, it is unclear whether one or more of these modifications are important for function. To examine the relationship between ubiquitination and function, we first measured the activities of all single Tat Lys-to-Arg mutants (Fig. 5a) and found that only mutation of Lys28 or Lys41 significantly inhibited Tat activity, in agreement with previous reports^13^. However, because multiple lysines can serve as acceptor sites (Fig. 4a, b), we reasoned that there may be redundancy among ubiquitination sites such that no single mutation eliminates Tat function. To limit this possible masking effect, we utilized the lysine-free Tat ΔK background and restored individual lysines, observing that only Lys41 recovered activity (Fig. 5b). This result highlights that Lys41 is required to create a proper structural framework, but even in this case activity was still 2.5-fold lower than wild type Tat, indicating that other lysines also are important. We next restored individual lysines within the Lys41-only background and found that Lys28 recovered the most activity (Fig. 5c), supporting the Lys-to-Arg mutant data (Fig. 5a). To fully recover function, we introduced the remaining seven lysines individually to the Tat ΔK (+K41, +K28) background and found, surprisingly, that any one of three different lysines (at positions 12, 51, or 85) was sufficient to restore wild type activity (Fig. 5d). Thus, Tat requires just three lysines for full function, and the location of the presumed ubiquitination site is flexible. Interestingly, all of the three-lysine Tat variants show near wild type levels of ubiquitination (Fig. 5e) even though just three of these sites preferentially restore activity. It is worth noting that adding Lys28 to the Lys41-only background recovers some ubiquitination (Fig. 5e, compare lanes 2 and 3), suggesting that Lys28 may be acetylated or ubiquitinated, analogous to the overlapping modifications of two lysines in Smad7^56^. Further supporting flexible acceptor site usage, two different Tat triple lysine-to-arginine mutants retained robust ubiquitination and wild type levels of transcriptional activity (Supplementary Fig. 8).

Finally, we used the more sensitive viral spreading assay to examine these lysine requirements in the context of HIV-1 replication in CD4+ Jurkat cells, which express considerable PJA2 (Supplemental Fig. 3b). Tat lysine mutants were cloned into the Nef locus, which is dispensable for viral replication in tissue culture, of a fully replication-competent HIV-1 NL4-3 clone. As expected, the lysine-free Tat ΔK virus was completely replication incompetent (Fig. 5f) whereas both Tat ΔK (+K41) and Tat ΔK (+K41, +K28) displayed slower replication kinetics than wild type. The Tat ΔK (+K41) virus in particular, which contains no modifiable lysines, never reached the peak titer of wild type virus, even at 10 days post infection. Notably, two different three-lysine Tat viruses replicated faster than the two-lysine Tat ΔK (+K41, +K28) virus, corroborating both that the third, presumably ubiquitinated, lysine results in a more active Tat and that its precise location is flexible. These viral replication experiments underscore an even more pronounced phenotype for the ubiquitinated Tat lysines.

### Tat enhances the interaction between PJA2 and P-TEFb

**Figure 6:**
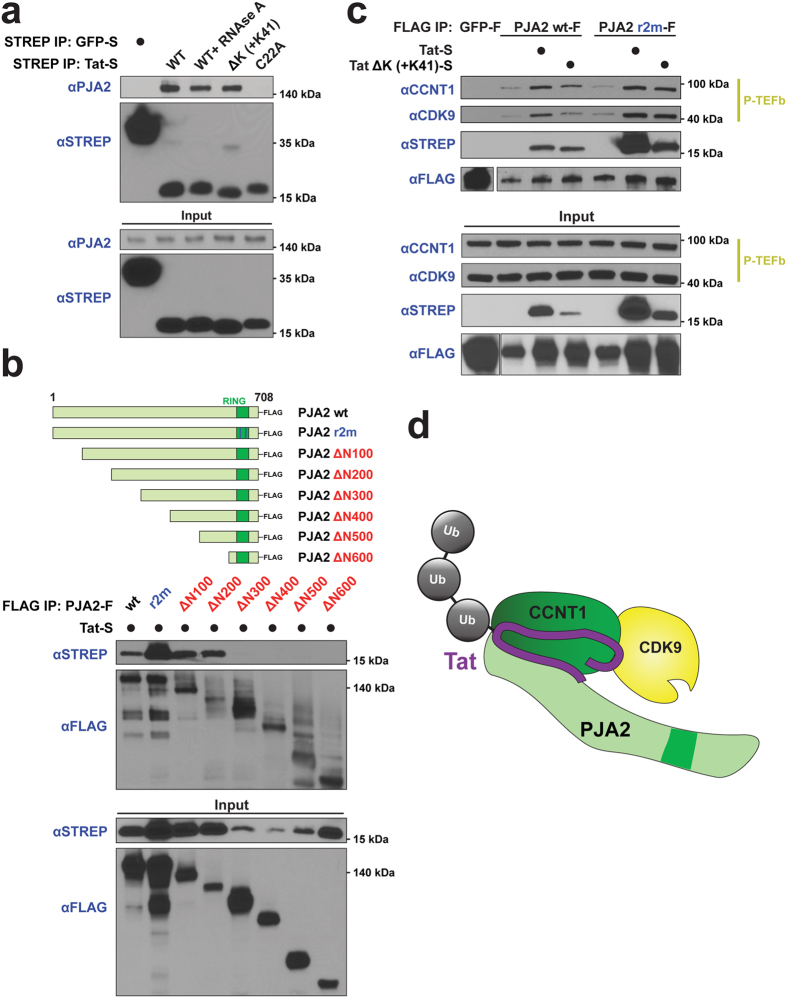
Tat enhances the interaction of PJA2 with P-TEFb. (**a**) STREP-tagged GFP, wild type Tat, or Tat mutants were transfected into HEK 293T cells and a STREP IP was used to detect an interaction with endogenous PJA2 by Western blotting with anti-PJA2 antibody. Cropped PJA2 blots are shown. For un-cropped images, see Sup. Fig. 9. (**b**) (top) Domain representation of FLAG-tagged PJA2 mutants used to map the Tat interacting region. (bottom) FLAG-tagged wild type PJA2, r2m, or truncation mutants were co-transfected with Tat-STREP (Tat-S) into HEK 293T cells. A FLAG IP of PJA2 variants was used to assess the interaction with Tat. (**c**) FLAG-tagged GFP, PJA2 wild type or r2m expression plasmids were co-transfected with vector, Tat-STREP or Tat ΔK (+K41)-STREP into HEK 293T cells. A FLAG IP elution was probed with anti-STREP, anti-CYCT1, and anti-CDK9 to detect PJA2 interactions with Tat and P-TEFb. FLAG blots are from the same exposure but are cropped for presentation. Cropped blots are shown for all antibodies. For un-cropped images, see Sup. Fig. 9. (**d**) Model of the PJA2-P-TEFb-Tat complex. PJA2 ubiquitinates Tat in the context of P-TEFb predominantly with Lys27 and Lys29 polyubiquitin chains. Tat also enhances the interaction between PJA2 and P-TEFb.

Given that the Tat-P-TEFb complex is the preferred substrate for PJA2, we examined the interactions of these proteins in more detail. Transiently transfected wild type Tat strongly precipitated endogenous PJA2 in an RNA-independent manner (Fig. 6a). Importantly, Tat C22A, an unfolded Tat mutant unable to interact with P-TEFb^13^, did not pull down PJA2, supporting that Tat folded in the P-TEFb complex is the preferred substrate for PJA2 (Fig. 3f). Additionally, Tat ΔK (+K41), which is not detectably ubiquitinated (Fig. 5e), interacted with PJA2 similarly to wild type Tat, demonstrating that Tat does not need ubiquitin acceptor sites to interact with its ligase.

We next mapped the interaction determinants in PJA2 using a series of N-terminal truncation mutants. Unexpectedly, the full-length but catalytically inactive PJA2 r2m pulled down substantially more Tat than wild type PJA2 (Fig. 6b), likely due to r2m expression also stabilizing Tat protein levels (Fig. 6b, input lane 2). This stabilization of Tat is supported by the observation that Tat protein levels decreased with siRNA knockdown of PJA2 (Fig. 2b). The Tat-interaction region of PJA2 maps between amino acids 200–300, highly conserved between human and mouse, and different from the 400–530 or 530–630 regions that interact with other protein partners^38^,^39^,^45^. Like the result with r2m, binding of the truncation mutants also correlates with Tat stabilization. Given that Tat complexed with P-TEFb is the preferred ubiquitination substrate for PJA2 in the cell (Fig. 3d,e), we also examined the interaction of PJA2 with P-TEFb. Transiently transfected PJA2-FLAG pulled down P-TEFb, and, to our surprise, the co-expression of Tat dramatically increased the recovery of CCNT1 and CDK9 subunits in the wild type and r2m PJA2-FLAG precipitate (Fig. 6c). These data further support a novel signaling axis composed of a Tat-PJA2-P-TEFb complex (Fig. 6d) that controls a currently undefined step in the transcription pathway.

## Discussion

Considerable effort has been dedicated to identifying the cellular protein complexes hijacked by HIV to complete its life cycle. HIV infection is currently incurable as the virus integrates into the human genome, and, depending on the cellular context, can enter a “latent” state of infection. In this latent state, the virus remains stably integrated, yet minimal or no viral RNA is transcribed, preventing clearance and resulting in a persistent infection^57^. The HIV Tat protein controls viral gene expression and, therefore, provides an excellent framework to understand mechanisms that regulate and activate latency^58^. Of particular importance, then, are the host proteins that Tat co-opts to induce transcription.

Here we report on the novel function of the RING finger ubiquitin ligase PJA2 to ubiquitinate Tat and control viral transcription. PJA2 has been shown to induce the degradative ubiquitination of core and regulatory components of several kinase complexes^38^,^39^,^45^,^46^. This is the first report of non-degradative ubiquitination for this ligase. Further, we show that PJA2 specifically targets Tat bound to P-TEFb to generate diverse, atypical polyubiquitin linkages through Lys27, Lys29, and Lys33. It is intriguing that Tat ubiquitination has the signature of a degradative mark, as it is targeted by a typically degradative ligase and the site specificity of ubiquitination is low. Similar to the other known PJA2 substrates, Tat functions as a regulator of a kinase complex (P-TEFb) and even stimulates the interaction between PJA2 and P-TEFb. One exciting possibility is that Tat rewired degradative ubiquitination by PJA2 into a positive signaling function. Ubiquitination appears to stabilize Tat protein levels as PJA2 co-expression increases Tat protein accumulation while PJA2 knockdown destabilizes Tat. This stabilization requires Tat acceptor lysines, as the Tat ΔK (+K41) mutant, which is undetectably ubiquitinated (Fig. 5e), is not stabilized as strongly as wild type Tat by PJA2 co-expression (Fig. 6c, STREP input) even though both proteins interact comparably with PJA2 (Fig. 6a,c IP), supporting a role for ubiquitination in the stabilization phenotype. PJA2 may therefore act to delay the transition to latency by increasing the half-life of Tat molecules. In addition to its own ubiquitination, Tat may even employ PJA2 to regulate the kinase activity of CDK9. Future work will uncover the amount of cross talk between the kinase and ubiquitin signaling pathways in HIV transcription.

HIV encodes a small 9 kb RNA genome with limited coding capacity. However, the virus has found many solutions to maximize the information contained in its genome, such as using an adaptable Rev monomer to assemble a higher order oligomeric RNA export complex^59^. We believe that the post-translational ubiquitination of Tat represents another viral strategy of genetic multi-tasking. In this case, one lysine residue encodes at least two possible protein states, modified and unmodified, each with an ability to execute a distinct function. Ubiquitination in combination with other known Tat post-translational modifications would allow a single viral protein to generate a large regulatory network to finely control transcription. The observation that re-introducing Lys28 restores substantial ubiquitination (Fig. 5e, lane 3) demonstrates that one amino acid can even encode three states (unmodified, acetylated^16^, ubiquitinated). It is unclear at the moment how Tat transitions through these three states to activate transcription. Our current work suggests a model where Tat first binds P-TEFb, which creates a substrate for ubiquitination by PJA2. This ubiquitinated Tat species might facilitate the formation of another protein-protein complex (e.g. the super elongation complex^36^) required for transcription. As Lys28 acetylation is eventually required for TAR binding, any ubiquitin mark on Lys28 would need to be removed. Interestingly, the YOD1 DUB, which is known to preferentially cleave Lys27, Lys29, and Lys33 di-ubiquitin chains^60^, substantially cleaves Tat polyubiquitin chains, demonstrating the presence of cellular machinery that could remove this atypical modification and regulate the transition between modified states. While a lysine deacetylase dedicated to specifically removing Tat Lys28-Ac is not known, it is likely that this modification is also dynamically regulated to allow coordinated rounds of modification of a single lysine residue. It will also be interesting to see if post-translational modifications at different sites (e.g. Lys28 and Lys50) act in an exclusive or combinatorial manner.

Compared to other transcription factors, Tat is a relatively small protein. The covalent attachment of a polyubiquitin chain drastically increases Tat’s effective size, generating a large signaling scaffold with multiple protein-protein interaction surfaces. At the moment, however, it is unclear which proteins in the Tat transcriptional circuit possess ubiquitin-binding domains. Determining which proteins can read the ubiquitin mark would help uncover the exact output of the ubiquitin signal. CDK9 has been shown to be ubiquitinated by the SKP2 ligase^61^, resulting in an increased affinity of the Tat-TAR-P-TEFb complex. In this case, it is uncertain whether the ubiquitin moiety itself contributes to the increased affinity or whether a protein with a ubiquitin-binding domain is recruited to CDK9-Ub to stimulate the affinity. In any case, identifying “readers” of the ubiquitin mark more generally is an exciting area of research.

The lysine mutant reporter data (Fig. 5a–d) highlight a striking result in which a Tat protein with different combinations of only three lysines is fully functional. However, there is a clear epistasis to this plasticity, as a functional Tat strictly requires Lys41, which helps it adopt its structure by folding on the CCNT1 scaffold via a set of hydrogen bond interactions^52^. A plastic ubiquitination mark, as detailed here, can then be layered over this partially folded scaffold. The functional data are supported by conservation analysis, which shows that Lys28 and Lys41 are the two most conserved lysines in the Tat activation domain^62^. The additional ubiquitinated lysines appear to tolerate mutation since multiple lysines can fully substitute as the acceptor site. These results are reminiscent of the plasticity of Tat for TAR RNA recognition, where a single arginine within a background of lysines could substitute for its arginine-rich motif when located at two different positions^63^. The virus appears to have encoded Tat in a way that balances strict amino acid requirements with relaxed positional requirements for distinct functions, thereby producing a protein that is more resilient to the high mutation rate of the virus.

It has become increasingly clear that HIV-1 interacts extensively with ubiquitin conjugation pathways during the course of infection. Degradative ubiquitin signaling is hijacked by the Vif protein, which recruits the cellular cytidine deaminase APOBEC3G to a CBFβ-Elongin B/C-Cullin 5 ubiquitin ligase for cleavage^64^,^65^, and the Vpu protein, which assembles a βTRCP-SKP1-Cullin1 complex to target the cytoplasmic domain of the CD4 receptor for ubiquitin-dependent degradation^66^,^67^. A non-degradative, signaling function has been shown for Gag, where monoubiquitination of the p6 domain contributes to proper budding of the virus^68^. Here we demonstrate an example of non-degradative ubiquitination for the Tat protein by the host E3 ligase, PJA2. The incredible diversity of modifiers (>600 E3 ligases in mammalian cells^21^), modified states (monoubiquitin, polyubiquitin with diverse linkages), and signaling outputs (degradation, re-localization, altered protein-protein interactions) makes the ubiquitin modification system an attractive target for an invading virus to hijack in order to rewire the cellular environment for sustained infection. Future research will uncover how the remaining ubiquitin signaling proteins identified here also contribute to regulating viral transcription.

## Methods

### Cell culture, plasmids, reagents

HeLa cells and derived cell lines (HeLa^provirusΔtat^, HeLa^LTR:FFL^ 11) (ATCC, CCL-2) and HEK 293T cells (ATCC, CRL-3216) were maintained in DMEM (10% FBS, 1% pen/strep) at 37 °C with 5% CO_2_. Jurkat E6-1 (ATCC, TIB-152) and Sup-T1 (ATCC, CRL-1942) cells were maintained in RPMI (10% FBS, 1% pen/strep) at 37 °C with 5% CO_2_. All proteins were cloned into pcDNA4-TO (Thermo) unless otherwise noted. Ubiquitin signaling proteins were cloned with a C-terminal 3x-FLAG tag, the HIV-1 Tat protein (HXB2 isolate) was cloned with a C-terminal 2x-STREP tag, and human ubiquitin was cloned with an N-terminal HA tag. All point mutants were generated by standard quick change mutagenesis. The Tat ΔK and ubiquitin ΔK mutants were ordered as a gBlock gene fragment from IDT. PJA2 truncation mutants were generated by PCR and cloned with a C-terminal 3xFLAG tag vector. DNA was transfected with Poly Jet (Signa Gen) or PEI and siRNA was transfected with HiPerFect (Qiagen) according to the manufacturer’s protocols. MG132 was purchased from Selleckchem (S2619). 2x and 4x Laemmli sample buffer were purchased from BioRad. siRNA was purchased as a FlexiPlate from Qiagen.

### Antibodies

The HA antibody (MMS-101P) was purchased from Covance. The PJA2 antibody (A302-991A) was purchased from Bethyl. The CCNT1 (sc-8127), CDK9 (sc-8338), and Tat (sc-65913) antibodies were purchased from Santa Cruz Biotechnology. The STREP-HRP antibody (71591) was purchased from Millipore. The FLAG antibody (F1804) was purchased from Sigma. The GAPDH antibody (AM4300) was purchased from Ambion.

### Cloning, expression, and purification of recombinant proteins

Full length PJA2 with a C-terminal 6x His-tag, Tat (1–86) with a C-terminal 2x STREP tag, human ubiquitin and mutants (ΔK, K6^only^, K11^only^, K27^only^, K29^only^, K33^only^, K48^only^ and K63^only^) with an N-terminal HA tag were cloned into the pGEX6p-1 vector (GE Healthcare) and expressed as a fusion protein with an N-terminal GST tag. The fusion proteins were expressed in *E. coli* BL21 (DE3). The GST-fusion proteins were digested on column with PreScission protease. The released ubiquitin proteins were collected and further purified by Superdex 200 Increase (GE Healthcare). The released PJA2 and Tat were further purified by nickel affinity chromatography or Strep-Tactin affinity purification (IBA), respectively. CDK9 (7–332) with a 6x His-tag and cyclin T1 (1–266) were cloned into pFastBac Dual vector, and HIV-1 Tat (1–86) was cloned into the pFastBac1 vector. The complex of CDK9/cyclin T1/Tat was expressed and purified as previously described 52. Human E1 and UbcH5b protein were a gift from the lab of John Gross.

### Targeted RNAi screen of high confidence Tat interactors

HeLa cells were infected with a VSV-G pseudo-typed HIV virus deleted for the Env, Tat, and Nef proteins by start site mutations. GFP was cloned in the Nef locus, which is dispensable in cell culture, to facilitate clonal selection. One clone with a single integrant, HeLa^provirusΔtat^, was used for the screen. Cells were seeded in a 96-well plate and transfected with non-silencing (N.S.) or host-specific siRNA to obtain 5 nM-10 nM final concentration using HiPerFect. All siRNA sequences are listed in Supplemental Table S1. After 48 h, cells were transfected with 0.5 ng of a Tat-expressing plasmid and a CMV-firefly luciferase plasmid as a transfection and toxicity control using PEI. Care was taken to transfect Tat-expressing plasmid in the linear range of activation. After an additional 48 hours, cells were lysed in cell lysis buffer (5 mM Tris-HCl pH 8.0, 85 mM KCl, 0.5% IGEPAL CA-630). Cell-associated HIV p24 production was detected by p24 ELISA. In parallel, 10 uL of lysate was used in a standard luciferase assay. All p24 values were normalized to firefly luciferase activity for every siRNA to generate a relative Tat activity score, defined as [p24^host RNAi^/FFL^host RNAi^]/[p24^N.S. RNAi^/FFL^N.S. RNAi^]. For analysis of protein knockdown, RNAi cells were lysed with 2x Laemmli sample buffer followed by SDS-PAGE/Western blotting with specific antibodies.

### *In vitro* ubiquitination assay

*In vitro* ubiquitination assays were performed with purified recombinant human E1 (0.1 μM), recombinant human UbcH5b (0.2 μM), 0.1 μM E3 (full length PJA2 or mutant r2m), 5 μM HA-tagged ubiquitin (wild type or mutants) and 0.3 μM Tat or Tat/CCNT1/CDK9 complex in reaction buffer containing 50 mM Tris-HCl pH 7.4, 100 mM NaCl, 1 mM DTT, 5 mM MgCl_2_, 4 mM ATP at 37 °C. Reactions were quenched by addition of 4x Laemmli sample buffer with b-mercaptoethanol before loading onto a gel for SDS-PAGE. Ubiquitination of Tat was verified by immunoblotting with anti-STREP or anti-Tat antibody, as indicated.

### Denaturing *in vivo* ubiquitination assay

HEK 293T or HeLa cells were seeded in 10 cm dishes and transfected with 7 μg total DNA containing STREP-tagged bait protein and HA-Ubiquitin with Poly Jet. After 30 hours, cells were lysed in an equal volume SDS Lysis Buffer (2% SDS, 50 mM Tris-HCl pH 8.0, 150 mM NaCl, 0.5 mM DTT) and boiled. After boiling, SDS Dilution Buffer (1% SDS, 50 mM Tris-HCl pH 8.0, 150 mM NaCl, 0.5 mM DTT) was added to samples, which were then sonicated and centrifuged. Cleared supernatant was incubated with Streptactin Agarose for 3 hours at room temperature. The Streptactin resin was then washed 4x with RIPA buffer (1% Triton X-100, 50 mM Tris-HCl pH 8.0, 150 mM NaCl, 0.5% Na-deoxycholate, 0.1% SDS) at room temperature. Bound STREP-tagged protein was eluted with 1x STREP elution buffer (IBA-GmbH). Ubiquitination of immunoprecipitated material was detected by anti-HA Western. For RNAi-ubiquitination experiments, cells were first transfected with siRNA for 48 hours prior to DNA transfection. For MG132 treatment, cells that had been transfected with DNA for 24 hours were then incubated with 2.5 μM MG132 for an additional 16 hours before harvesting.

### RNA elongation assay

HeLa^provirusΔtat^ cells seeded at 40% confluence were transfected with siRNA to obtain 10 nM final concentration. After 48 hours, cells were transfected −/+a Tat-expressing plasmid for an additional 10 hours. RNA was purified on Zymo Quick-RNA Mini Prep columns, DNAse-treated with TURBO DNAse (Thermo), and used in RT-qPCR reactions with proximal (Prox F 5′-CTCTCTGGTTAGACCAGATCTGA-3′, Prox R 5′-GTGGGTTCCCTAGTTAGCCAGA-3′) and distal (Dist F 5′-CACATGAAGCAGCACGACTT-3′, Dist R 5′-GGTCTTGTAGTTGCCGTCGT-3′) HIV primer sets. All values were normalized to the abundance of RPLP0 mRNA.

### Transcriptional reporter assay

We used a previously characterized HeLa cell line containing an integrated copy of the HIV LTR driving the expression of firefly luciferase^11^. Reporter assays were carried out as previously described^13^. Briefly, HeLa^LTR:FFL^ cells were transfected with 0.25 ng of wild type or mutant Tat-expressing plasmid with PolyJet. 0.25 ng of CMV-Renilla luciferase was included as a control for transfection efficiency. Cells were lysed in 1x Lysis Buffer (Promega) and firefly and Renilla luciferase activities were measured with the Promega DualGlo luciferase system. Different quantities of Tat plasmid were initially transfected to determine the linear range of activity. 100 ng of pcDNA4-HA-Ub plasmid was used in ubiquitin mutant overexpression activity assays.

### Identification of ubiquitinated Tat lysines by MS

HEK 293T cells were seeded in a 15 cm cell culture dish and transfected with 2.5 ug of Tat-expressing plasmid by PolyJet. After 48 hours, a denaturing STREP IP was performed on the lysate. The eluate was run on a gel for SDS-PAGE, and a band corresponding to ubiquitinated Tat (~25 kDa) was excised from the gel and digested with trypsin in gel as previously reported^69^,^70^. Samples were analyzed on a Thermo Scientific Orbitrap Elite MS system. Peptides were then separated by an organic gradient from 5% to 30% ACN in 0.1% formic acid. Data were analyzed by the Protein Prospector analysis suite^71^. Data were searched against the HIV Tat protein sequence (Uniprot accession P04608) concatenated with a HIV Tat protein sequence that was randomized. Fully tryptic peptides only were considered, with up to 2 missed cleavage sites. A static modification for carbamidomethyl cysteine was specified as well as variable modifications for oxidation of methionine, acetylation of the protein N-terminus, and Gly-Gly modification of lysines to capture tryptic ubiquitin remnant peptides. Spectra of interest were manually validated.

### HIV spreading infection in Jurkat cells

The pNL4-3 proviral vector was used to generate all viruses. Tat mutants were cloned into the Nef locus of the virus with engineered SacII/XbaI sites. A codon-swapped variant of Tat was used to generate all mutant alleles to prevent recombination with the endogenous Δtat sequence. Wild type and mutant Tat viruses were generated by transfection of pNL4-3 constructs into HEK 293T cells for 48 hours. Supernatant was collected, and viruses were purified and titered by p24 ELISA. For the spreading infection, Jurkat cells were seeded in a 96 well plate. Next day, cells were infected with wild type and mutant Tat viruses by spinoculation at an MOI of 0.5. A day 0 time point was taken after spinoculation. Supernatant was removed every 2-3 days and lysed 1:1 with PBS-2% Triton X-100. Jurkat cells were split 1:1 when reaching high cell density. The supernatant p24 was analyzed by ELISA.

### Affinity purification and Western blotting

HEK 293T or HeLa cells at 60% confluence were transfected with STREP-tagged or FLAG-tagged bait plasmid using PolyJet. After 40 hours, cells were lysed in Lysis Buffer (50 mM Tris-HCl pH 8.0, 150 mM NaCl, 2 mM MgCl_2_, 0.5% Triton X-100, 0.2% Na-deoxycholate, Roche protease inhibitor cocktail) for 30 minutes at 4 °C. Lysate was centrifuged and added to Streptactin Superflow resin or magnetic M2-FLAG resin and rotated for 3 hours at 4 °C. The resin was washed 4x with WASH buffer (50 mM Tris-HCl pH 8.0, 150 mM NaCl, 2 mM MgCl_2_, 0.5% Triton X-100). Washed resin was eluted with 1x STREP elution buffer or FLAG elution buffer (200 μg/mL 3x FLAG peptide, diluted in WASH buffer) at room temperature for 30 minutes. The eluate was mixed 1:1 with 2x Laemmli sample buffer (BioRad), boiled for 5 minutes, and loaded on a SDS-PAGE gel. Proteins were detected with specific antibodies.

### Deubiquitination assay

The assay was carried out using the UbiCREST Deubiquitinase Enzyme Kit (Boston Biochem). HEK 293 cells seeded in a 15 cm cell culture dish were transfected at 60% confluence with 15 μg DNA containing STREP-tagged Tat, FLAG-tagged PJA2 and ubiquitin by the Poly Jet transfection reagent. After 30 hours, cells were harvested, lysed in Lysis Buffer and rocked for 30 minutes at 4 °C. Lysate was centrifuged and supernatant was incubated with Streptactin Superflow resin slurry (IBA-GmBH) for 3 hours at 4 °C. The resin was washed sequentially with high salt WASH buffer (Phosphate-Buffered Saline +1 M NaCl +0.05% Tween-20) for 4 times, PBS-T (Phosphate-Buffered Saline +0.05% Tween-20) for 2 times, and DUB reaction buffer (1 ml) for 2 times, which was supplied with the kit, and eventually resuspended in DUB buffer (total volume was 450 μl). Resin was distributed equally among 9 microcentrifuge tubes on ice and 45 μl of DUB reaction buffer was added. The reactions were started by adding 5 μl DUB enzyme or 5 μl DUB buffer (control reaction) separately, followed by mixing and incubation at 37 °C for 30 min. After the reaction, collected resin was mixed with 50 μL of 1× Laemmli sample buffer (containing 2-Mercaptoethanol) and boiled before loading onto SDS-PAGE gel. Ubiquitination of Tat was verified by immunoblotting with anti-Tat antibody.

### Statistical Analysis

Data are expressed as mean −/+SEM. An unpaired, two-tailed Student’s t-test was used to evaluate statistical significance. A p-value < 0.05 was considered statistically significant.

## Additional Information

**How to cite this article:** Faust, T. B. *et al*. PJA2 ubiquitinates the HIV-1 Tat protein with atypical chain linkages to activate viral transcription. *Sci. Rep.* **7**, 45394; doi: 10.1038/srep45394 (2017).

**Publisher's note:** Springer Nature remains neutral with regard to jurisdictional claims in published maps and institutional affiliations.

## Supplementary Material

Supplementary Information
