# Supplementary material for: PJA2 ubiquitinates the HIV-1 Tat protein with atypical chain linkages to activate viral transcription
Source: Sci Rep. 2017 Mar 27;7:45394. doi: 10.1038/srep45394 (PMC5366948; doi:10.1038/srep45394)
Supplement: Supplementary Information [file srep45394-s1.pdf]

PJA2 ubiquitinates the HIV-1 Tat protein with atypical chain linkages to activate viral transcription

Tyler B. Faust[1], Yang Li[2], Gwendolyn M. Jang[3], Jeffrey R. Johnson[3], Shumin Yang[4,5], Amit Weiss[2], Nevan J. Krogan[3], Alan D. Frankel[2*]

[1]Tetrad Program, Department of Biochemistry and Biophysics, University of California, San Francisco, San Francisco, California

[2]Department of Biochemistry and Biophysics, University of California, San Francisco, San Francisco, California

[3]Department of Cellular and Molecular Pharmacology, University of California, San Francisco, San Francisco, California

[4]Department of Bioengineering and Therapeutic Science, University of California, San Francisco, San Francisco, California

[5]School of Medicine, Tsinghua University, Beijing, China

*Corresponding author

E-mail: frankel@cgl.ucsf.edu (ADF)

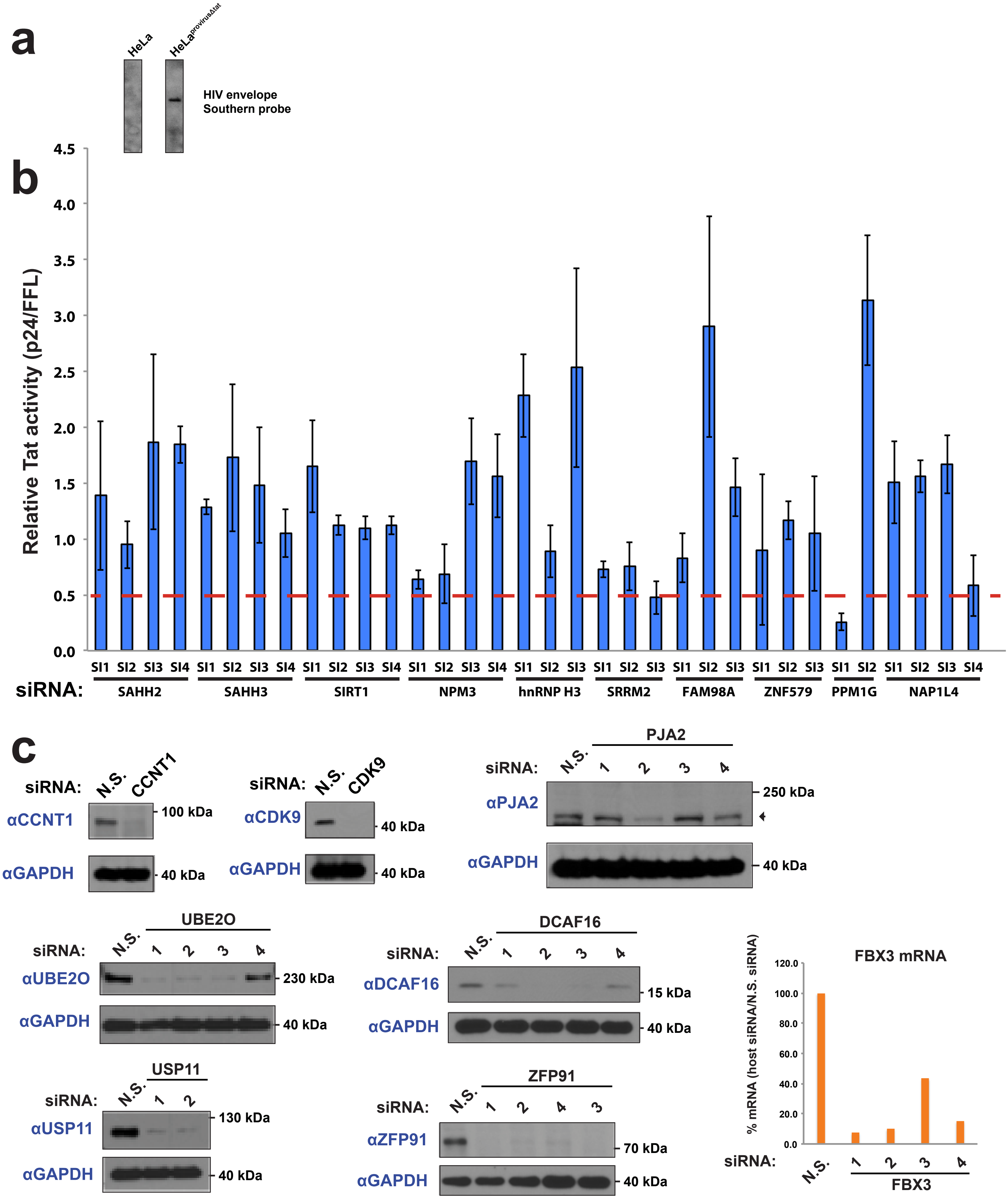
a
HeLa
HeLa provirusΔtat
HIV envelope Southern probe
b
Relative Tat activity (p24/FFL)
4.5
4.0
3.5
3.0
2.5
2.0
1.5
1.0
0.5
0.0
SI1 SI2 SI3 SI4 SI1 SI2 SI3 SI4 SI1 SI2 SI3 SI4 SI1 SI2 SI3 SI4 SI1 SI2 SI3 SI1 SI2 SI3 SI1 SI2 SI3 SI1 SI2 SI3 SI1 SI2 SI1 SI2 SI3 SI4
siRNA:
SAHH2
SAHH3
SIRT1
NPM3
hnRNP H3
SRRM2
FAM98A
ZNF579
PPM1G
NAP1L4
c
siRNA: N.S. CCNT1
αCCNT1
100 kDa
αGAPDH
40 kDa
siRNA: N.S. CDK9
αCDK9
40 kDa
αGAPDH
40 kDa
PJA2
siRNA: N.S. 1 2 3 4
αPJA2
250 kDa
αGAPDH
40 kDa
UBE2O
siRNA: N.S. 1 2 3 4
αUBE2O
230 kDa
αGAPDH
40 kDa
DCAF16
siRNA: N.S. 1 2 3 4
αDCAF16
15 kDa
αGAPDH
40 kDa
FBX3 mRNA
% mRNA (host siRNA/N.S. siRNA)
120.0
100.0
80.0
60.0
40.0
20.0
0.0
siRNA: N.S. 1 2 3 4
FBX3
USP11
siRNA: N.S. 1 2
αUSP11
130 kDa
αGAPDH
40 kDa
ZFP91
siRNA: N.S. 1 2 4 3
αZFP91
70 kDa
αGAPDH
40 kDa

Sup. Fig. 1

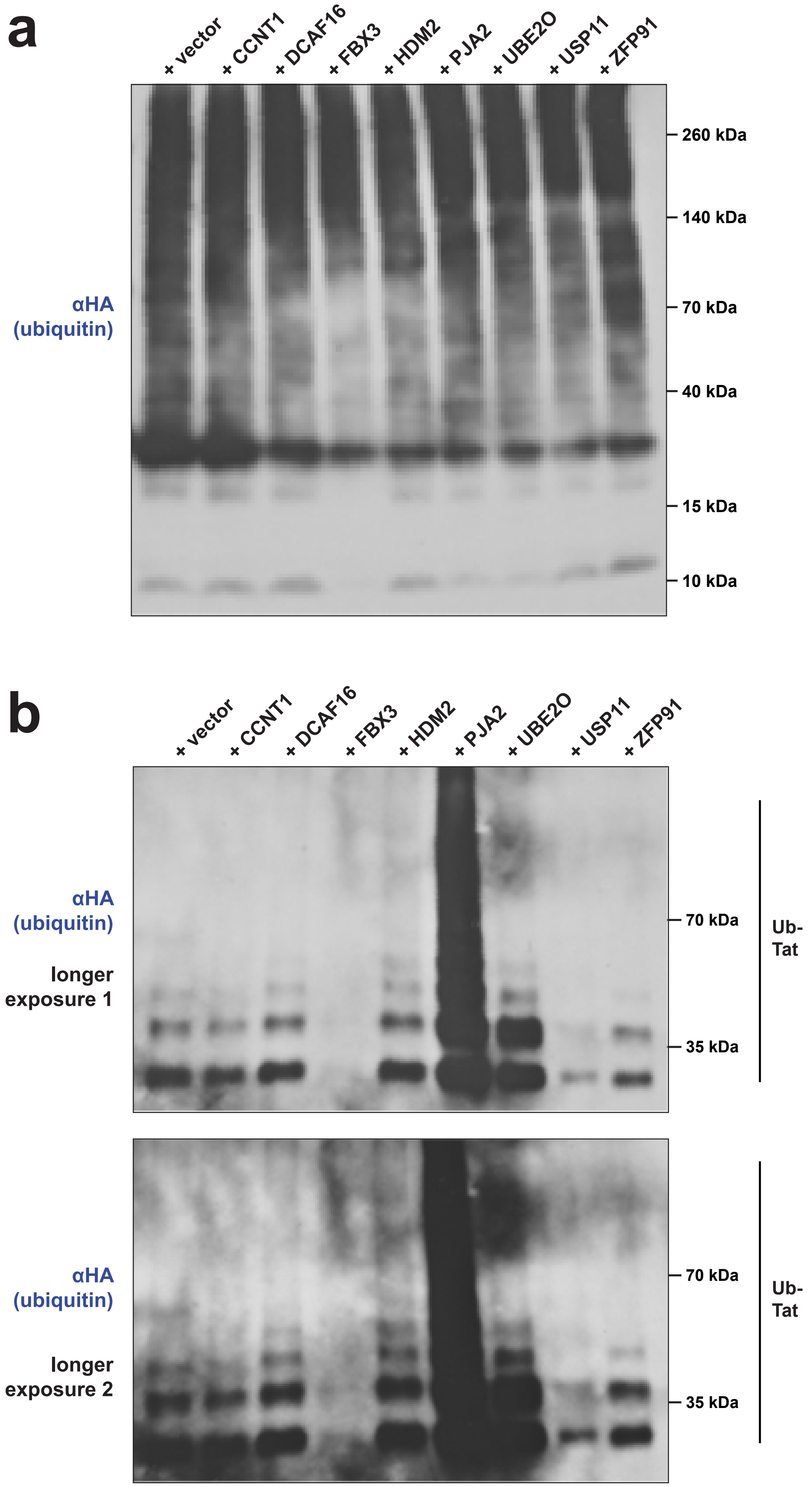

a
+ vector
+ CCNT1
+ DCAF16
+ FBX3
+ HDM2
+ PJA2
+ UBE2O
+ USP11
+ ZFP91
αHA
(ubiquitin)
260 kDa
140 kDa
70 kDa
40 kDa
15 kDa
10 kDa
b
+ vector
+ CCNT1
+ DCAF16
+ FBX3
+ HDM2
+ PJA2
+ UBE2O
+ USP11
+ ZFP91
αHA
(ubiquitin)
longer
exposure 1
70 kDa
35 kDa
Ub-
Tat
αHA
(ubiquitin)
longer
exposure 2
70 kDa
35 kDa
Ub-
Tat

Sup. Fig. 2

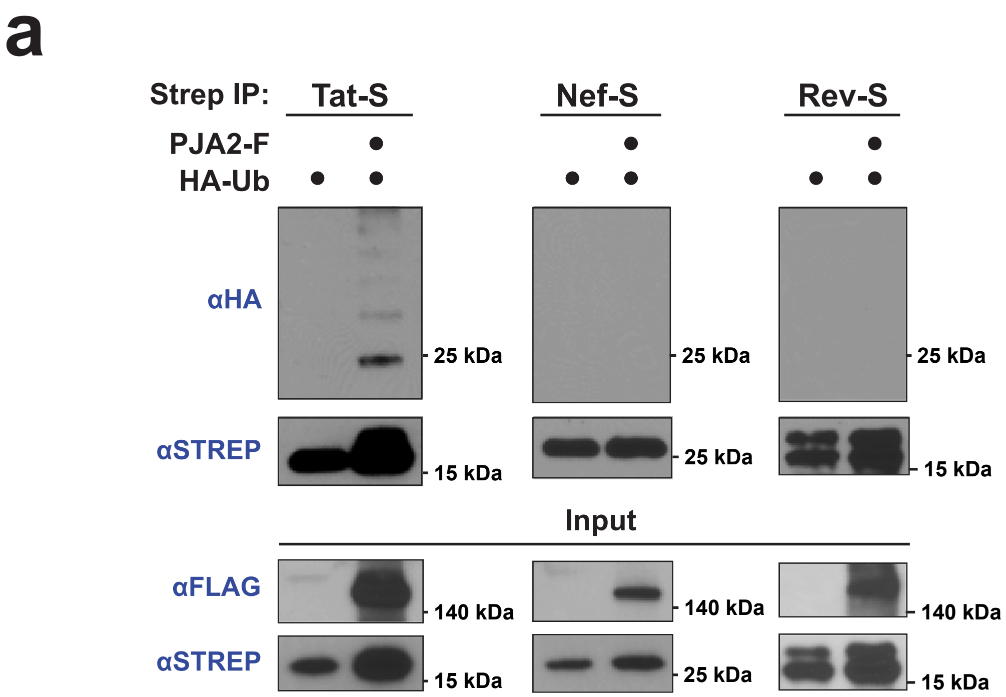
a
Strep IP:
Tat-S
Nef-S
Rev-S
PJA2-F
HA-Ub
αHA
25 kDa
αSTREP
15 kDa
25 kDa
Input
αFLAG
140 kDa
αSTREP
15 kDa
25 kDa

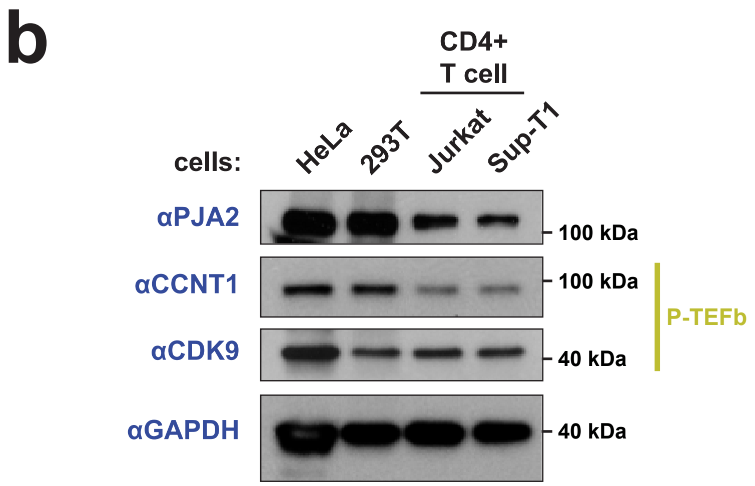
b
CD4+
T cell
cells:
HeLa
293T
Jurkat
Sup-T1
αPJA2
100 kDa
αCCNT1
100 kDa
αCDK9
40 kDa
αGAPDH
40 kDa
P-TEFb

Sup. Fig. 3

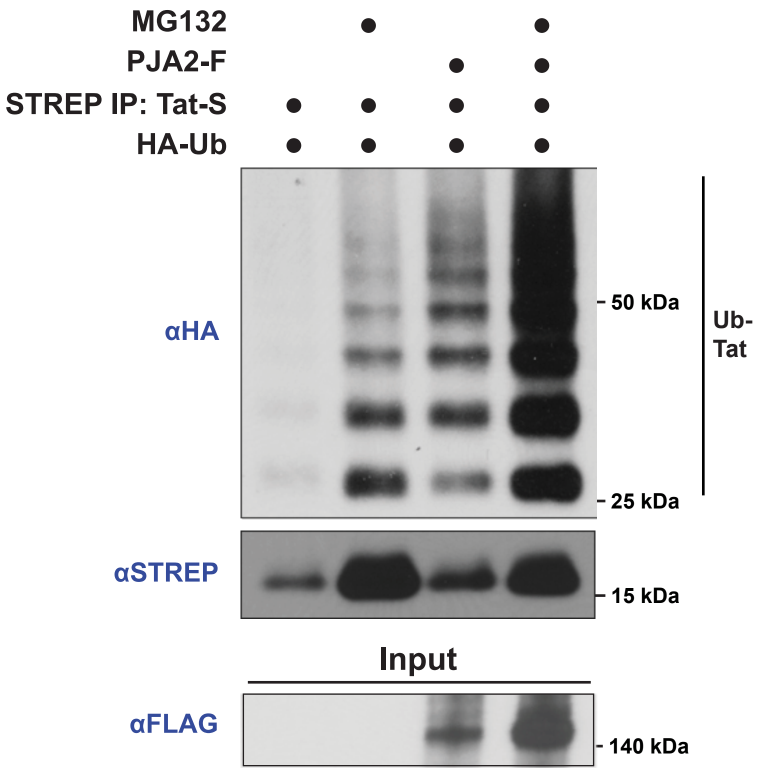
MG132
PJA2-F
STREP IP: Tat-S
HA-Ub
αHA
50 kDa
25 kDa
Ub-Tat
αSTREP
15 kDa
Input
αFLAG
140 kDa

Sup. Fig. 4

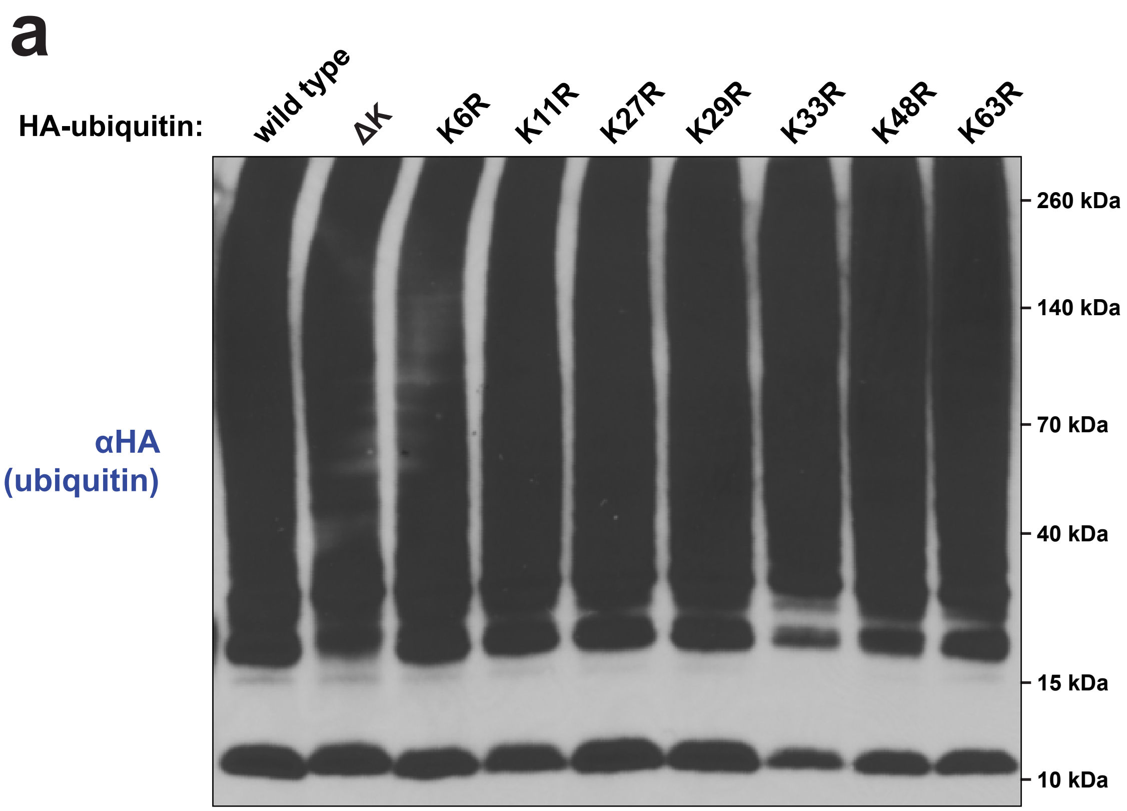
a
HA-ubiquitin:
wild type
ΔK
K6R
K11R
K27R
K29R
K33R
K48R
K63R
αHA
(ubiquitin)
260 kDa
140 kDa
70 kDa
40 kDa
15 kDa
10 kDa

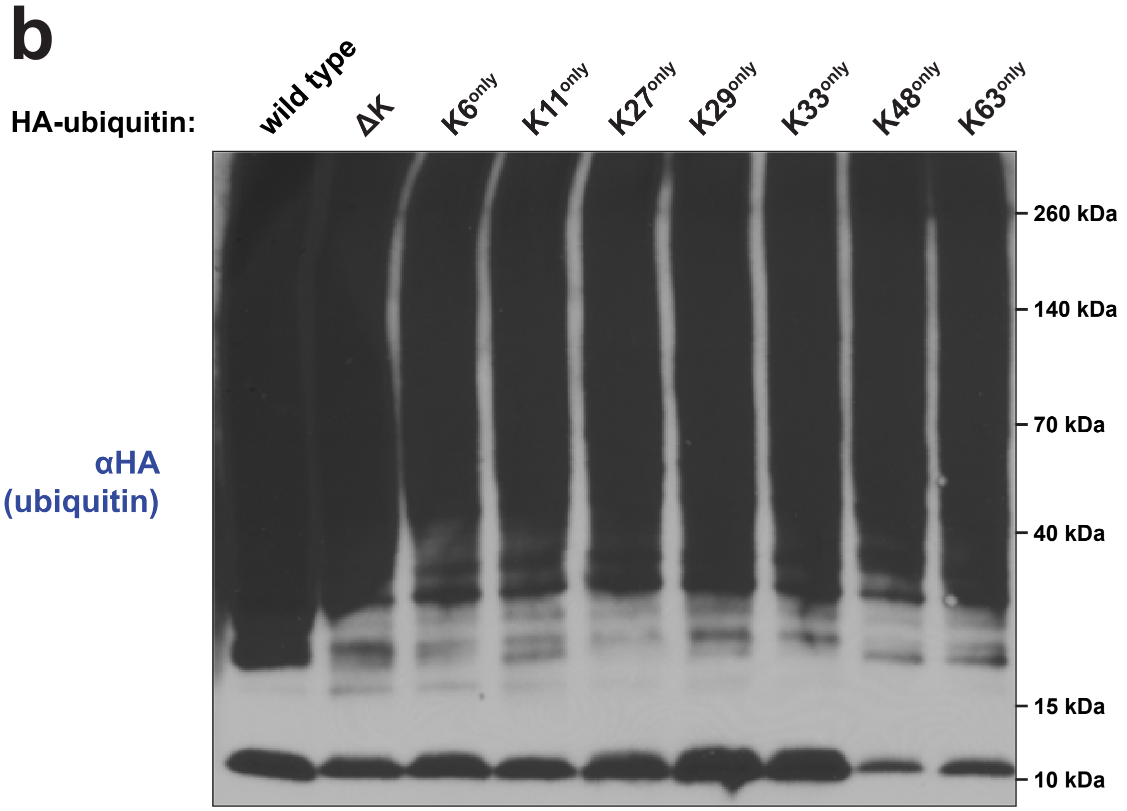
b
HA-ubiquitin:
wild type
ΔK
K6only
K11only
K27only
K29only
K33only
K48only
K63only
αHA
(ubiquitin)
260 kDa
140 kDa
70 kDa
40 kDa
15 kDa
10 kDa

Sup. Fig. 5

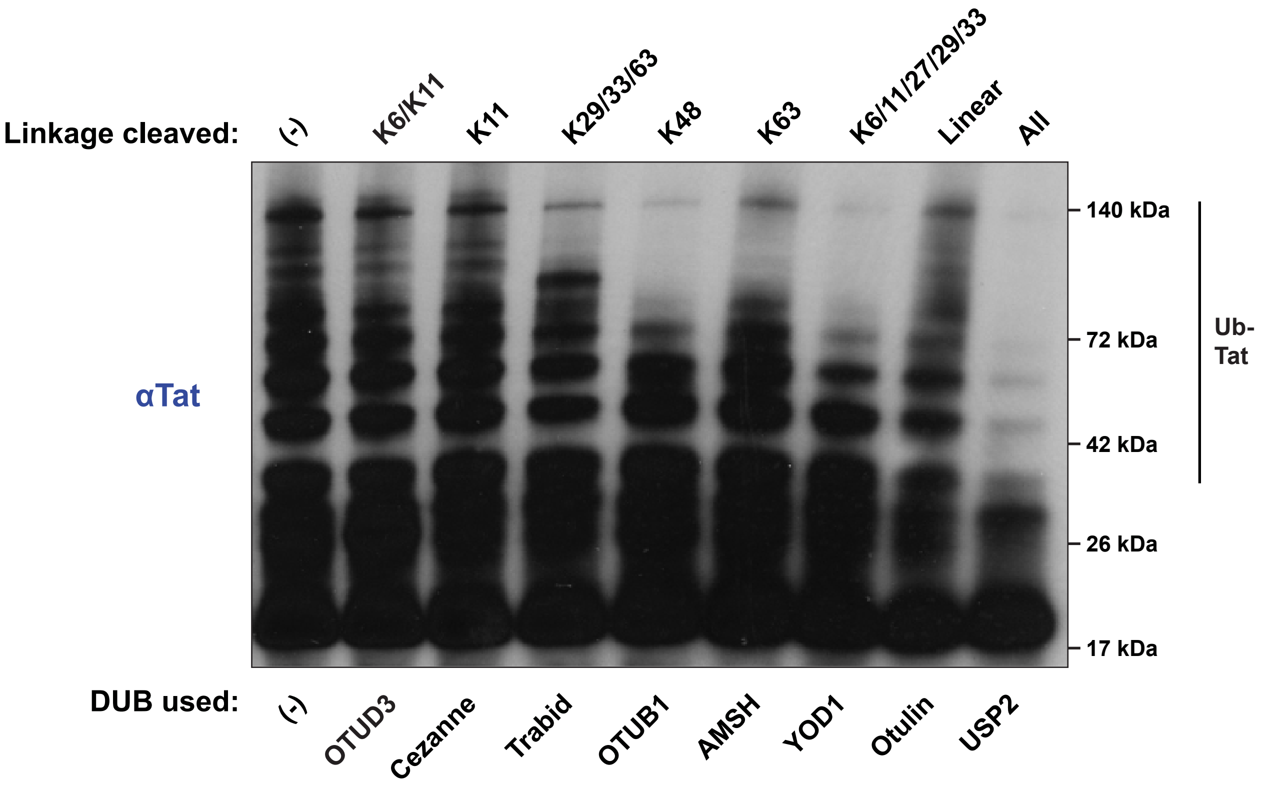

Linkage cleaved:
(-)
K6/K11
K11
K29/33/63
K48
K63
K6/11/27/29/33
Linear
All
αTat
140 kDa
72 kDa
42 kDa
26 kDa
17 kDa
Ub-
Tat
DUB used:
(-)
OTUD3
Cezanne
Trabid
OTUB1
AMSH
YOD1
Otulin
USP2

Sup. Fig. 6

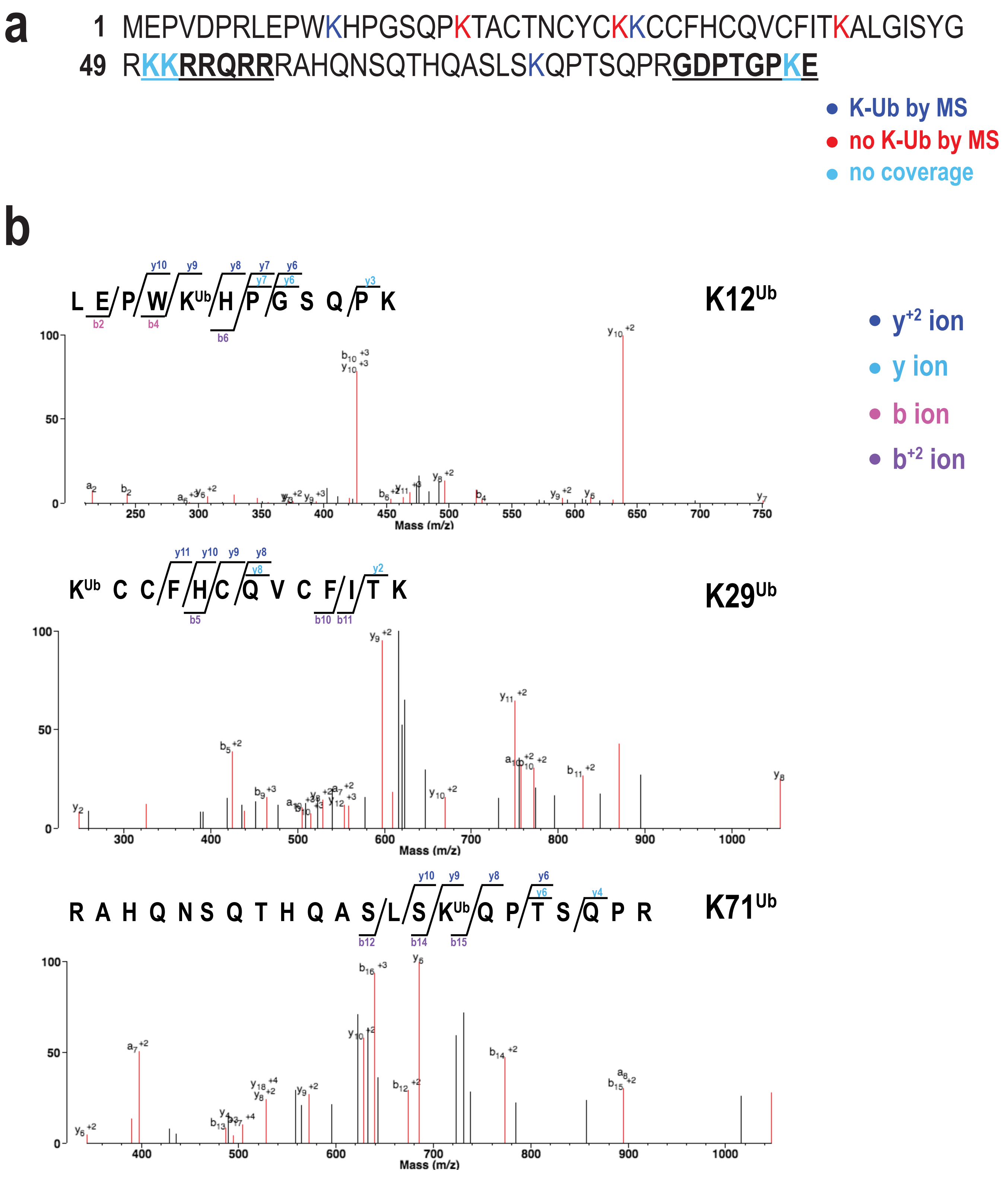
a
1 MEPVDPRLEPWKHPGSQPKTACTNCYCKKCCFHCQVCFITKALGISYG
49 RKKRRQRRRAHQNSQTHQASLSKQPTSQPRGDPTGPKE
K-Ub by MS
no K-Ub by MS
no coverage
b
L E P W K^Ub H P G S Q P K
K12^Ub
y^+2 ion
y ion
b ion
b^+2 ion
K^Ub C C F H C Q V C F I T K
K29^Ub
R A H Q N S Q T H Q A S L S K^Ub Q P T S Q P R
K71^Ub
Mass (m/z)
Mass (m/z)
Mass (m/z)

Sup. Fig. 7

a

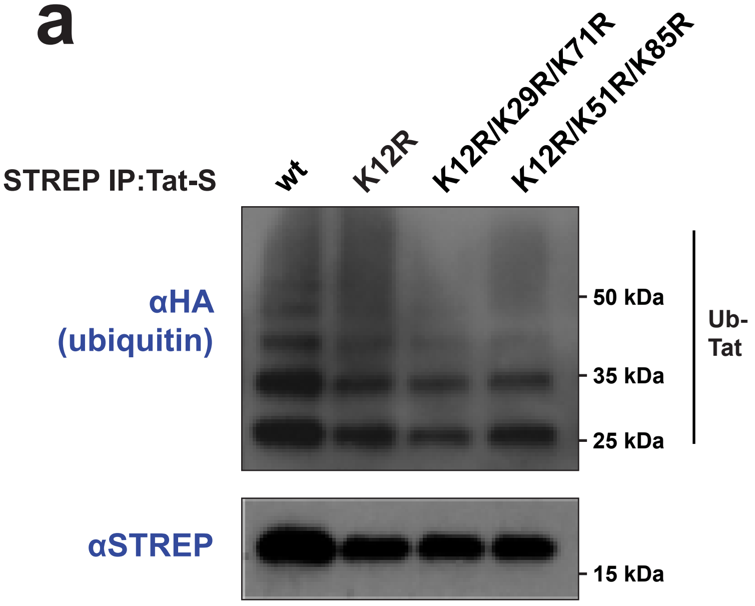
STREP IP:Tat-S
wt
K12R
K12R/K29R/K71R
K12R/K51R/K85R
αHA
(ubiquitin)
50 kDa
35 kDa
25 kDa
Ub-
Tat
αSTREP
15 kDa

b

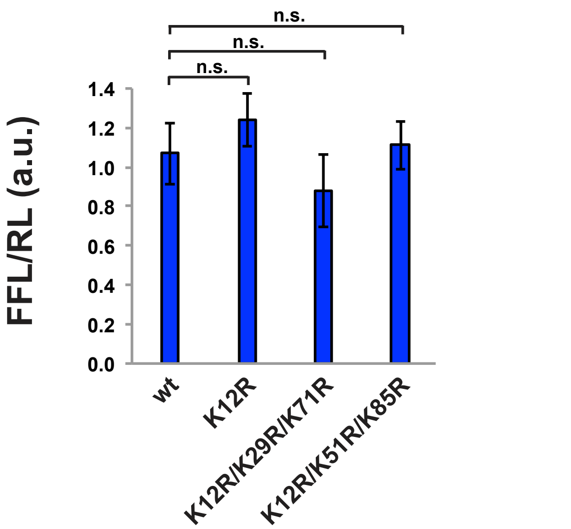
n.s.
n.s.
n.s.
FFL/RL (a.u.)
1.4
1.2
1.0
0.8
0.6
0.4
0.2
0.0
wt
K12R
K12R/K29R/K71R
K12R/K51R/K85R

Sup. Fig. 8

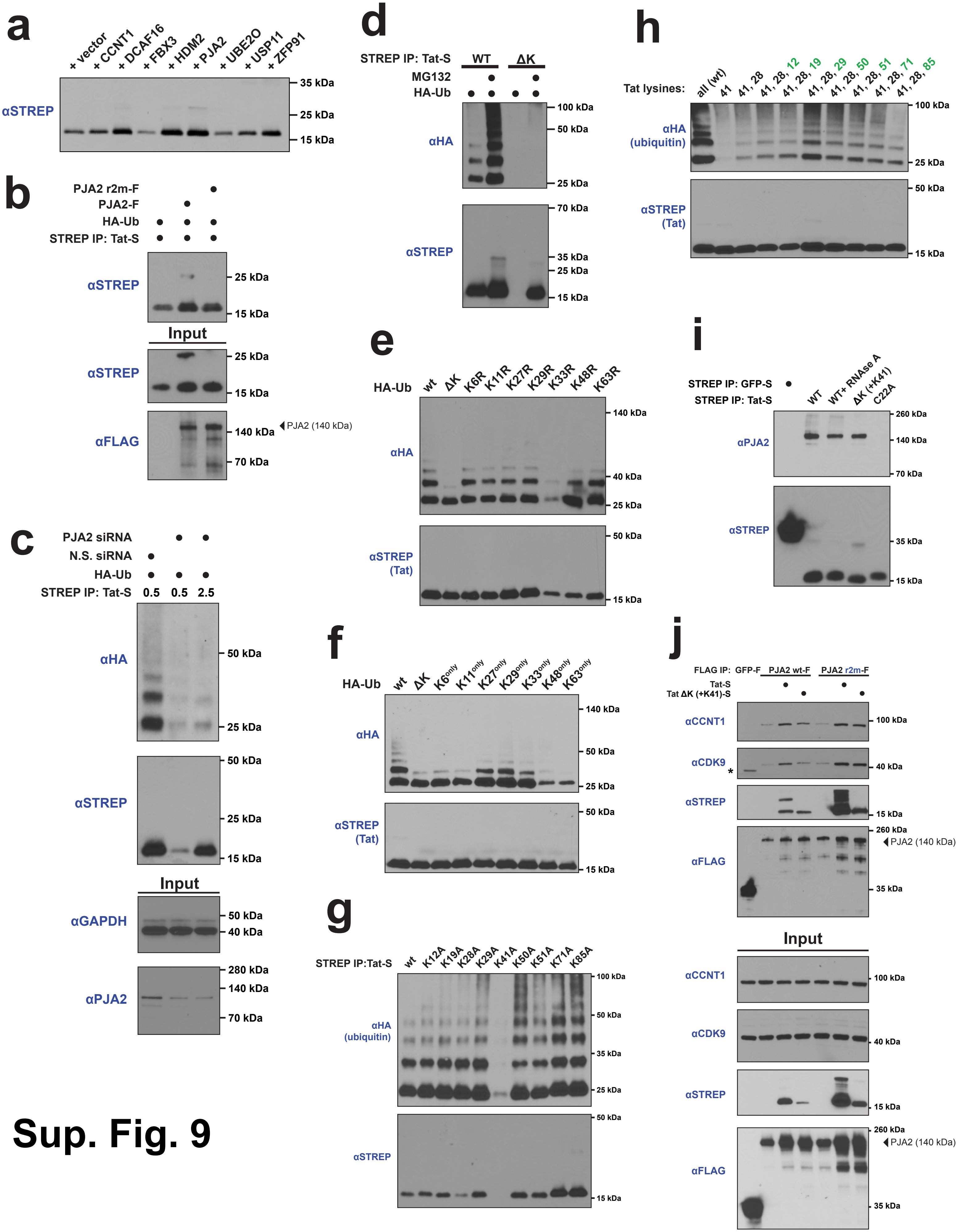
a
αSTREP
+ vector
+ CCNT1
+ DCAF16
+ FBX3
+ HDM2
+ PJA2
+ UBE2O
+ USP11
+ ZFP91
35 kDa
25 kDa
15 kDa
b
PJA2 r2m-F
PJA2-F
HA-Ub
STREP IP: Tat-S
αSTREP
Input
αFLAG
PJA2 (140 kDa)
140 kDa
70 kDa
c
PJA2 siRNA
N.S. siRNA
HA-Ub
STREP IP: Tat-S
0.5
2.5
αHA
αGAPDH
αPJA2
50 kDa
40 kDa
280 kDa
d
STREP IP: Tat-S
WT
ΔK
MG132
100 kDa
e
HA-Ub
wt
K6R
K11R
K27R
K29R
K33R
K48R
K63R
αSTREP (Tat)
f
K6only
K11only
K27only
K29only
K33only
K48only
K63only
g
STREP IP:Tat-S
K12A
K19A
K28A
K29A
K41A
K50A
K51A
K71A
K85A
αHA (ubiquitin)
h
Tat lysines:
all (wt)
41
41, 28
41, 28, 12
41, 28, 19
41, 28, 29
41, 28, 50
41, 28, 51
41, 28, 71
41, 28, 85
i
STREP IP: GFP-S
WT
WT+ RNAse A
ΔK (+K41)
C22A
260 kDa
j
FLAG IP: GFP-F
PJA2 wt-F
PJA2 r2m-F
Tat-S
Tat ΔK (+K41)-S
αCCNT1
αCDK9
*

Sup. Fig. 9

Entrez Gene Id	NCBI gene symbol	siRNA Target Sequence	Product Id	Product Name	Gene Description
9867	PJA2	CTGGCAAGTTCAGATACTAAA	SI00108325	Hs_PJA2_1	praja ring finger 2, E3 ubiquitin protein ligase
9867	PJA2	TTGGCACATATGAGGCTTAAA	SI00108332	Hs_PJA2_2	praja ring finger 2, E3 ubiquitin protein ligase
9867	PJA2	ATCCACCATACTCAAGAGTTA	SI00108339	Hs_PJA2_3	praja ring finger 2, E3 ubiquitin protein ligase
9867	PJA2	TAAGGTTAGTAAAGCATACAA	SI00108346	Hs_PJA2_4	praja ring finger 2, E3 ubiquitin protein ligase
1025	CDK9	TAGGGACATGAAGGCTGCTAA	SI00605066	Hs_CDK9_5	cyclin-dependent kinase 9
5496	PPM1G	CAGGACCTGAGGACTCAACTA	SI02658684	Hs_PPM1G_6	protein phosphatase, Mg2+/Mn2+ dependent, 1G
5496	PPM1G	CCAGAGGATGAAGTAGAACTA	SI02658691	Hs_PPM1G_7	protein phosphatase, Mg2+/Mn2+ dependent, 1G
10768	AHCYL1	CAGGGTGGTAAAGCTAAATGA	SI00090328	Hs_AHCYL1_1	adenosylhomocysteinase-like 1
10768	AHCYL1	AAACAGTTGTATCGTATGCAA	SI00090335	Hs_AHCYL1_2	adenosylhomocysteinase-like 1
10768	AHCYL1	CTGATAGAACTCTATAATGCA	SI00090342	Hs_AHCYL1_3	adenosylhomocysteinase-like 1
10768	AHCYL1	CCCACTTGGATTTATAGTATA	SI00090349	Hs_AHCYL1_4	adenosylhomocysteinase-like 1
26273	FBXO3	CTGACGATTATCGATGTTCAT	SI00097783	Hs_FBXO3_1	F-box protein 3
26273	FBXO3	CTGCCAGAACTTAGCTCTGTA	SI00097797	Hs_FBXO3_3	F-box protein 3
26273	FBXO3	CAGGTCGGGTATATGAATACA	SI03071999	Hs_FBXO3_5	F-box protein 3
26273	FBXO3	CTGGTTTACCTCTTATGTCAA	SI03100202	Hs_FBXO3_6	F-box protein 3
63893	UBE2O	TCCCGGGACCATTCCATGGAA	SI04153863	Hs_UBE2O_2	ubiquitin-conjugating enzyme E2O
63893	UBE2O	AACCAGATCATCCTGAAGCTA	SI04228679	Hs_UBE2O_3	ubiquitin-conjugating enzyme E2O
63893	UBE2O	TGGCACGGTGATCGACGTCAA	SI04280017	Hs_UBE2O_4	ubiquitin-conjugating enzyme E2O
63893	UBE2O	TGGCCTGTCGACCACGTTATA	SI04346699	Hs_UBE2O_5	ubiquitin-conjugating enzyme E2O
54876	DCAF16	CGGGAGATCTGCCGAGGATAA	SI04162718	Hs_C4orf30_1	DDB1 and CUL4 associated factor 16
54876	DCAF16	TCTGACTAAATCAGCCTATAA	SI04163901	Hs_C4orf30_2	DDB1 and CUL4 associated factor 16
54876	DCAF16	CCAGATGTTCTTGACAGCTCA	SI04284903	Hs_C4orf30_3	DDB1 and CUL4 associated factor 16
54876	DCAF16	ATGGGCCACAAGCATACTTTA	SI04373012	Hs_C4orf30_4	DDB1 and CUL4 associated factor 16
8237	USP11	CTGCGTCGGGTACGTGATGAA	SI02780813	Hs_USP11_5	ubiquitin specific peptidase 11
8237	USP11	ACCGATTCTATTGGCCTAGTA	SI02781156	Hs_USP11_6	ubiquitin specific peptidase 11
23382	AHCYL2	CAGCGGCTTCATATACAGATA	SI00110467	Hs_KIAA0828_1	adenosylhomocysteinase-like 2
23382	AHCYL2	CAGGTTCTAAACTCTATATAT	SI00110474	Hs_KIAA0828_2	adenosylhomocysteinase-like 2
23382	AHCYL2	CAACATCTATTCCACTCTCAA	SI00110481	Hs_KIAA0828_3	adenosylhomocysteinase-like 2
23382	AHCYL2	AGGCAACACCAGCAAATTATA	SI00110488	Hs_KIAA0828_4	adenosylhomocysteinase-like 2
23411	SIRT1	CAAGCGATGTTTGATATTGAA	SI00098434	Hs_SIRT1_1	sirtuin 1
23411	SIRT1	CAGGATTATTGTATTTACGTT	SI00098441	Hs_SIRT1_2	sirtuin 1
23411	SIRT1	TTGGGTCTTCCCTCAAAGTAA	SI00098448	Hs_SIRT1_3	sirtuin 1
23411	SIRT1	AGCCATCGGAATGTTAAATTA	SI04954068	Hs_SIRT1_8	sirtuin 1
10360	NPM3	CACCCGCTCCTTCACCTTTAA	SI00661122	Hs_NPM3_2	nucleophosmin/nucleoplasmin 3
10360	NPM3	CCAGATTGTTACGATGAGCAA	SI00661129	Hs_NPM3_3	nucleophosmin/nucleoplasmin 3
10360	NPM3	CCCGGTCACTATGGACAGTTT	SI04142824	Hs_NPM3_5	nucleophosmin/nucleoplasmin 3
10360	NPM3	AAAGACGAGTGTAATGTGGTA	SI04278176	Hs_NPM3_6	nucleophosmin/nucleoplasmin 3
4676	NAP1L4	AAGAAAGTATGCAGCGCTATA	SI03127383	Hs_NAP1L4_5	nucleosome assembly protein 1-like 4
4676	NAP1L4	CTGCGGGTCACCTCATATTTA	SI03208758	Hs_NAP1L4_7	nucleosome assembly protein 1-like 4
4676	NAP1L4	GTGGACATGCTGAGTGAATTA	SI04194309	Hs_NAP1L4_8	nucleosome assembly protein 1-like 4
4676	NAP1L4	TTCGCTGTGGCTCGTCCTTAA	SI04288228	Hs_NAP1L4_9	nucleosome assembly protein 1-like 4
80829	ZFP91	CTGCGGCACACTTATCTTCAA	SI05109223	Hs_ZFP91_9	ZFP91 zinc finger protein
80829	ZFP91	CAGCTCTTAAAGTGAGGGTTA	SI05109230	Hs_ZFP91_10	ZFP91 zinc finger protein
80829	ZFP91	GTGGATTTACTTGTCGACAAA	SI05150971	Hs_ZFP91_14	ZFP91 zinc finger protein
80829	ZFP91	CCGCGACTCCTATGCATAGAA	SI05150985	Hs_ZFP91_16	ZFP91 zinc finger protein
3189	HNRNPH3	AGCGACCGGGACCATATGATA	SI03144295	Hs_HNRPH3_5	heterogeneous nuclear ribonucleoprotein H3 (2H9)
3189	HNRNPH3	ATCGCCTGACAGGCATTTAAA	SI04141858	Hs_HNRPH3_7	heterogeneous nuclear ribonucleoprotein H3 (2H9)
3189	HNRNPH3	AACATTGACGATGGACTACCA	SI04306736	Hs_HNRPH3_8	heterogeneous nuclear ribonucleoprotein H3 (2H9)
163033	ZNF579	CCGCGAAGCCTTCGCCACCAA	SI00776979	Hs_ZNF579_1	zinc finger protein 579
163033	ZNF579	CCCGGTTTGCAGAATGGAGGA	SI00776986	Hs_ZNF579_2	zinc finger protein 579
163033	ZNF579	CCCAACATGTGTCTTAAGGCA	SI00777000	Hs_ZNF579_4	zinc finger protein 579
23524	SRRM2	CGCCACCTAAACAGAAATCTA	SI00733460	Hs_SRRM2_4	serine/arginine repetitive matrix 2
23524	SRRM2	CTCGACGAAGATCCCGGTCAA	SI04216898	Hs_SRRM2_6	serine/arginine repetitive matrix 2
23524	SRRM2	CTCGATCATCTCCGGAGCTAA	SI04173995	Hs_SRRM2_5	serine/arginine repetitive matrix 2
25940	FAM98A	TTGGAGTCGTTGGAAGATCTA	SI04209373	Hs_FAM98A_1	family with sequence similarity 98, member A
25940	FAM98A	AAACGTTTGGATGTCACTGTA	SI04225263	Hs_FAM98A_2	family with sequence similarity 98, member A
25940	FAM98A	CAGCCGAAACGTTCAGTCTTA	SI04315241	Hs_FAM98A_3	family with sequence similarity 98, member A
904	CCNT1	AGGCTTTGAACTAACAATTGA	SI02625707	Hs_CCNT1_5	cyclin T1

Supplemental Table S1

Supplemental Figure Legends

Supplementary Fig. 1. Additional relative Tat activity data from the targeted RNAi screen. (a) Southern blot of parental HeLa or HeLa$^{provirus\Delta tat}$ cell line with DIG-labeled HIV envelope. HeLa$^{provirus\Delta tat}$ contains a single integrated provirus that is responsive to Tat transfection and was used for the siRNA functional screen. (b) Normalized, relative Tat activity ([p24$^{host\ RNAi}$/FFL$^{host\ RNAi}$] / [p24$^{N.S.\ RNAi}$/FFL$^{N.S.\ RNAi}$]) for Tat-interacting host factors (SAHH2, SAHH3, SIRT1, NPM3, hnRNP H3, SRRM2, FAM98A, ZNF579, PPM1G, and NAP1L4) not shown in Fig. 1. Data are represented as the mean -/+ SEM of three biological replicates for independent siRNA transfections. The red dotted line represents activity cutoff for the screen. (c) Western blots or qPCR quantitations to demonstrate protein or RNA knockdown, respectively, for CCNT1, CDK9, or ubiquitin pathway proteins. The arrow next to the PJA2 blot indicates the expected size of the protein.

Supplementary Fig. 2. Tat host factors do not globally alter cellular ubiquitination. (a) Input anti-HA blot for experiment shown in Fig. 2a. (b) Longer exposures of anti-HA Western for IP experiment from Fig. 2a. These blots demonstrate that only PJA2 substantially alters Tat ubiquitination.

Supplementary Fig. 3. PJA2 specifically ubiquitinates the HIV Tat protein. (a) HeLa$^{LTR:FFL}$ cells were transfected with HA-Ub, vector or PJA2-F, and STREP-tagged Tat, Nef, or Rev. A denaturing STREP IP was used to detect viral protein ubiquitination by HA Western blotting. (b) Protein expression comparison across multiple cell lines. 100,000 cells of HeLa, HEK 293T, Jurkat, or Sup-T1 were

collected and lysed in SDS loading buffer. Western blots of whole cell lysate with specific antibodies demonstrate endogenous protein expression.

Supplementary Fig. 4. Tat protein levels are stabilized by proteasome inhibition. *In vivo* ubiquitination assay of Tat-STREP (Tat-S) co-transfected with vector or PJA2-F in HEK 293T cells, -/+ MG132 treatment.

Supplementary Fig. 5. Ubiquitin mutant expression. (a) Anti-HA (ubiquitin) Western blot of input from experiment in Fig. 3b. (b) Anti-HA (ubiquitin) Western blot of input from experiment in Fig. 3d. The blots demonstrate similar expression between wild type and mutant ubiquitin proteins.

Supplementary Fig. 6. Cleavage with linkage-specific de-ubiquitinating enzymes (DUBs) supports that Tat is modified with Lys27, Lys29, and Lys33 linkages. Tat-STREP purified from HEK 293T cells was used as the substrate for UbiCREST analysis. Shown is an anti-Tat blot after DUB treatment, demonstrating that YOD1 and OTUB1 decrease Tat polyubiquitination signal. Comparison of the YOD1 lane (cleaves Lys6, Lys11, Lys27, Lys29, and Lys33) with the OTUD3 lane (cleaves Lys6 and Lys11, no change in signal compared to control) indicates that YOD1 cleaves Lys27, Lys29, and Lys33 chains on Tat. USP2, which cleaves most known chain types, serves as a positive control DUB and indicates that the majority of the higher molecular Tat species on the blot are ubiquitinated.

Supplementary Fig. 7. Mass spectrometry identification of ubiquitinated Tat lysines. (a) 82.6% peptide coverage of the HXB2 Tat protein sequence. Underlined and bolded is the sequence not covered by any peptide for MS. Color coding reflects whether a lysine was modified with a ubiquitin di-glycine

tryptic remnant fragment, unmodified, or undetected. (b) Mass spectra for Tat peptides containing lysine-di-glycine ubiquitin remnant tryptic fragments for Lys12, Lys29, and Lys71. Identified y and b ions are labeled on each peptide sequence.

Supplementary Fig. 8. Tat contains multiple, redundant ubiquitin acceptor sites that support ubiquitination and transcriptional activity: (a) *In vivo* ubiquitination assay of STREP-tagged Tat (Tat-S) wild type and mutants purified from HEK 293T cells. The anti-HA Western detects ubiquitinated Tat. (b) Transcriptional reporter activities in the HeLa LTR:FFL reporter cell line for the Tat proteins from (a). Each Tat plasmid was co-transfected with CMV-RL as a transfection efficiency control. Activity values are reported as firefly luciferase activity normalized to Renilla luciferase. Data are plotted as the mean of biological triplicates, -/+ SEM. A two-tailed, unpaired Student's t-test was used to evaluate statistical significance. A p-value of <0.05 was considered significant, which was not obtained for any of the indicated comparisons (n.s.).

Supplementary Fig. 9: Un-cropped blots from main figures. (a) STREP blot from Fig. 2a IP. (b) STREP IP, STREP input and FLAG input blots from Fig. 2b, left panel. Expected size of PJA2-FLAG shown with arrow to right of blot. (c) HA and STREP blots from IP, GAPDH and PJA2 blots from input from Fig. 2b, right panel. (d) HA and STREP blots from IP from Fig. 3a. (e) HA and STREP blots from IP from Fig. 3b. (f) HA and STREP blots from IP from Fig. 3d. (g) HA and STREP blots from IP from Fig. 4a. (h) HA and STREP blots from IP from Fig. 5e. (i) PJA2 and STREP blots from IP from Fig. 6a. (j) CCNT1, CDK9, STREP and FLAG blots from IP and input from

Fig. 6c. Asterisk in CDK9 IP blot indicates non-specific band in GFP lane. Expected size of PJA2-FLAG shown with arrow to right of blot.

Supplemental Table S1: Sequences for siRNAs used in targeted RNAi screen.
